# The role of re‐presentation in the treatment of liquid expulsion

**DOI:** 10.1002/jaba.70052

**Published:** 2026-01-22

**Authors:** Emma M. Auten, Kathryn M. Peterson

**Affiliations:** ^1^ Children's Specialized Hospital–Rutgers University Center for Autism Research, Education, and Services (CSH–RUCARES) Somerset NJ USA; ^2^ Department of Pediatrics Rutgers Robert Wood Johnson Medical School New Brunswick NJ USA; ^3^ Department of Pediatrics School of Medicine, Emory University Atlanta GA USA; ^4^ Children's Healthcare of Atlanta Atlanta GA USA

**Keywords:** consecutive controlled case series, escape extinction, expel, feeding difficulties, re‐presentation

## Abstract

Children with feeding difficulties often engage in expulsion (i.e., spitting out) of liquid. Expulsion is problematic because it limits the volume of liquid that a child will consume. Researchers have used re‐presentation as an embedded component of escape extinction to treat expel. Although studies have demonstrated that re‐presentation can effectively reduce expel, it is unclear whether it should always be included with escape‐extinction‐based treatments. We describe a program evaluation project designed to examine the effects of re‐presentation on liquid expulsion for children with severe feeding difficulties. We conducted a prospective consecutive controlled case series to compare the effects of a function‐based treatment with and without re‐presentation and reported on the outcomes obtained for 17 children. Various patterns of responding emerged across participants. However, re‐presentation resulted in the greatest increases in mouth clean (i.e., swallowing) and lowest levels of expel for 10 participants. We discuss implications for research and practice.

Although eating and drinking are fundamental for growth and healthy development, many children experience feeding difficulties including avoidant/restrictive food intake disorder (American Psychiatric Association, 2013) and pediatric feeding disorder (Goday et al., 2019). Children with either diagnosis or with undiagnosed feeding difficulties are at risk for developing serious short‐ and long‐term negative consequences such as growth failure, malnutrition, various types of infection or illness, or impairments in cognitive and social functioning, to name a few (Volkert & Piazza, 2012). This is particularly concerning given the prevalence of pediatric feeding difficulties (Anil et al., 2019).

Eating and drinking represent complex behavior chains that begin with acceptance of food or liquid into the oral cavity and end with swallowing. Each response within this complex chain is critical for safe management of food and liquid and for retention (i.e., food or liquid remains inside the mouth and is swallowed). Children with disordered feeding histories may have missed opportunities to develop or refine these feeding skills, leading to problems with retention.

There is a wealth of empirical support for behavior‐analytic interventions as treatments for feeding difficulties and as tools to improve or teach feeding skills (Volkert & Piazza, 2012). Studies have focused on increasing acceptance of food and liquid (i.e., the first behavior in the chain) and decreasing inappropriate mealtime behavior (IMB; turning away from or pushing the food or liquid away; Williams & Seiverling, 2023). Research has predominantly shown that negative reinforcement, in the form of removal of the spoon or cup, is a factor that affects learned refusal behavior among children with feeding difficulties. Thus, escape extinction (i.e., nonremoval of the cup or spoon) is well established as an effective intervention (Volkert & Piazza, 2012; Williams & Seiverling, 2023). For children with severe and pervasive feeding difficulties who have been affected by serious medical or health‐related concerns, escape extinction may be a necessary treatment (Berth et al., 2019; Patel et al., 2002; Piazza et al., 2003).

Expulsion, defined across the literature as food or liquid exiting the mouth following acceptance (e.g., Ibañez, Peters, & Vollmer, 2021; LaRue et al., 2011; Wilkins et al., 2011), commonly co‐occurs with or emerges after a reduction in IMB among children with feeding difficulties. Although researchers have successfully assessed the function of IMB through the use of functional analysis (Piazza et al., 2003), no research to date has evaluated the function of expel. To test the effects of different consequences (e.g., tangible, attention) on expel, the bite or drink must be inside the mouth (i.e., expel is defined by food or liquid exiting the mouth). Tangible and attention test conditions could not be evaluated in isolation (i.e., without providing escape) unless the bite or drink was repeatedly re‐presented and deposited into the mouth following each occurrence of expel. Given that the topography of expel results in the bite or drink leaving the mouth (thereby providing escape from the food or liquid), researchers have hypothesized that expel is likely a member of the same functional response class as IMB (Sevin et al., 2002).

With this hypothesis, researchers have often treated expel with similar consequence‐based interventions as IMB (e.g., escape extinction, differential reinforcement for mouth clean [swallowing]; LaRue et al., 2011). Re‐presentation, during which the feeder replaces expelled solids or liquid into the child's mouth, is a well‐studied consequence‐based intervention (Allison et al., 2012; Coe et al., 1997; Sevin et al., 2002). Re‐presentation could lead to reductions in expel by breaking the response–reinforcer relation (i.e., removal of escape following expel) if the behavior is maintained by negative reinforcement. Re‐presentation could also serve as a teaching tool to aid in successful retention of liquid inside the mouth. In *Science and Human Behavior*, Skinner writes that a behavior may be differentially reinforced without specifically programmed contingencies through shaping that occurs with the “mechanical exigencies of the environment” (1965, p. 95). It is possible that re‐presentation increases the number of opportunities for the child to strengthen, refine, or better coordinate the oral and facial movements and muscles necessary for retention (e.g., tightening of the lips to retain fluid inside the oral cavity, coordination of the tongue to move liquid to the back of the mouth). By doing so, re‐presentation may provide repeated opportunities for the child's eating and drinking behavior (e.g., use of the tongue and teeth to hold the bolus inside the mouth, coordination of the tongue and soft palate to prevent spillage into the throat) to contact reinforcement.

Re‐presentation has been evaluated with solids (e.g., Wilkins et al., 2011) and liquids (Coe et al., 1997) and in conjunction with antecedent‐based arrangements (e.g., modified bite or drink placements, modified utensil presentations; Girolami et al., 2007; Ibañez, Peters, St Paul, et al., 2021; Sevin et al., 2002; Sharp et al., 2010, 2012; Wilkins et al., 2014). Few researchers have assessed the effects of re‐presentation during treatment of liquid refusal. This may be partially attributed to the fact that increasing consumption of a variety of healthy foods (rather than liquids) often serves as a primary goal of treatment. However, it is still important to evaluate methods for increasing liquid consumption. For many children with severe nutritional deficiencies, introducing a calorie‐dense and nutritionally complete liquid could offer substantial health benefits. Ibañez, Peters, and Vollmer (2021) compared re‐presentation with a modified chin prompt to treat liquid expulsion for three children with feeding difficulties. Ibañez, Peters, and Vollmer found that both treatments were effective in isolation; but for one participant, the combination of treatments was necessary for reduced expulsion. LaRue et al. (2011) implemented escape extinction without re‐presentation and still observed increases in mouth clean for solids and liquids; however, expels were not reported. Alternatively, Wilkins et al. (2011) found that liquid expulsion continued despite the inclusion of a re‐presentation procedure. Many providers and researchers embed re‐presentation as part of their escape‐extinction procedures (e.g., Allison et al., 2012; Berth et al., 2019), whereas others may introduce re‐presentation sequentially only after expulsion emerges (Sevin et al., 2002). It is not clear which route is optimal or whether expulsion would decrease with continued exposure to escape extinction alone (no re‐presentation in place), especially as the child acquires greater oral‐motor coordination and strength, as the child's food or liquid acceptance and consumption contact reinforcement, as food and liquid become less aversive (i.e., escape from the bite or drink is less valuable), or a combination. If not included with escape extinction, it may be difficult to determine when re‐presentation is warranted.

It is important to compare interventions with and without re‐presentation to determine its effects on liquid retention for children with complex and severe feeding difficulties. First, it is important to identify which components of treatment packages are necessary to achieve meaningful improvements in behavior. Re‐presentation is an intervention that requires precision and efficiency with implementation and may require additional training to conduct safely and correctly. Thus, the inclusion of unnecessary treatment components may result in additional time or resources being used. Second, clinicians should avoid the prescription of potentially contraindicated treatments. For example, re‐presentation could be contraindicated for a child who has a history of swallowing dysfunction as additional deposits of liquid inside the mouth could increase the risk of aspiration (Weir et al., 2007).

The purpose of the current study was to compare function‐based treatment involving escape extinction and reinforcement with and without re‐presentation to determine the relative effects of re‐presentation on expel and mouth clean with liquids. Treatment included additional function‐ or reinforcement‐based components (e.g., differential reinforcement, noncontingent access, attention extinction) for most participants. The study was carried out in an intensive day‐treatment program for children with severe feeding difficulties. As part of general clinical practice at the time of the study, this day‐treatment program typically included re‐presentation with escape extinction for certain clinical indications for use of this procedure that are equivalent to the inclusion criteria described below. Our goal was to conduct a clinical improvement evaluation, to evaluate the efficacy of re‐presentation as a component of a function‐based treatment, and to better understand the conditions under which the use of this component was warranted to refine clinical practice and streamline treatment. Thus, we conducted a prospective consecutive controlled case series study as a program evaluation project to evaluate this standard‐of‐care practice in our program (Hagopian, 2020). Children who met predefined clinical criteria to receive re‐presentation with escape extinction were included. These clinical data were then compiled for the current analysis. This evaluation allowed us to better understand beneficial and nonbeneficial effects of re‐presentation so that we could refine our clinical practice. The findings also may lead to important considerations for future research and clinical work.

## METHOD

### Participants, inclusion criteria, and informed consent

All children who participated in the study met criteria for a pediatric feeding disorder (Goday et al., 2019) or avoidant/restrictive food intake disorder (Estrem et al., 2025). All participants received previous less intensive outpatient services with little or no progress toward their feeding goals or had an emergent need for treatment services (e.g., impending tube placement, failure to thrive). The children's medical teams (e.g., primary care physicians, specialists such as pediatric gastroenterologists) referred them to the intensive program because the child's feeding difficulties had prevented them from progressing to age‐ or developmentally appropriate feeding routines and contributed to poor weight gain, growth concerns, serious health problems (e.g., malnutrition, dehydration), or a combination. Caregivers then pursued intensive treatment after referral.

Children were eligible for the clinical evaluation if (a) the child had a treatment goal to increase consumption (i.e., acceptance and mouth clean) of a nutritional liquid (e.g., PediaSure), (b) the child engaged in IMB or expel and did not accept and consume drinks at clinically acceptable levels in baseline, (c) the child had an identified escape function based on the results of a functional analysis of IMB, and (d) a provider with expertise in oral‐motor safety and a medical provider had confirmed the child was safe to participate in treatment. If any children displayed unexpected medical (e.g., vomiting, allergic reaction) or safety concerns (e.g., increase in wet vocalizations) during the evaluation, they would have been excluded; however, this did not occur. The clinical evaluation was conducted for a period of 2 years. There were 24 children who met the above criteria during the time of the clinical evaluation. We were unable to contact three caregivers to obtain consent for data sharing, and one other child's caregivers decided to pursue an alternative treatment progression. Of the remaining 20 children (see Table 1, Element 2), two children's raw data files were lost and one child's data (Mary) were not included in our analysis because team members did not conduct a reversal due to an error in planning (limiting experimental control for that data set). See Supporting Information A for Mary's data. This left 17 children's data to include in the analysis. Table 2 displays participant demographic information, their medical history related to their feeding difficulties, and the final treatment that was in place for each participant.

**TABLE 1:** Consecutive controlled case series reporting guideline checklist (Kaur et al., 2025).

**Element 1: A single‐case experimental design (SCED) is employed with each case**	
SCED(s) employed:	Multielement, Reversal
Independent Variables:	Escape extinction with re‐presentation and escape extinction without re‐presentation
Dependent Variables:	Expel per opportunity, mouth clean
Data Interpretation:	Graphs, tables
Treatment failures reported?	Yes
**Element 2: All consecutively encountered cases that underwent the procedure of interest or share a common characteristic are included when reporting outcomes**	
Number of consecutively encountered cases:	20
Number of responders to re‐presentation:	10
Number of responders to both treatments:	5
Number of responders to no re‐presentation only:	1
Number of nonresponders:	1
Number incomplete or drop out:	3
**Element 3: Criteria for selecting procedures and participants are described**	
Criteria for selecting procedures:	Escape function identified, clinical goal to increase liquid consumption
Criteria for selecting participants:	Enrolled in feeding program, cleared as safe oral feeder, medically stable
Assignment of participants to conditions:	All participants exposed to the same treatment conditions
Type of CCCS:	Prospective (Program Evaluation)
Randomization?	No
Group Design?	No
**Element 4: Findings are examined within and across participants in a manner that preserves the analysis of individual outcomes**	
Results portrayed at individual level?	Yes
Results analyzed across participants?	Yes
**Element 5: Multiple cases are included and well characterized**	
Setting variables reported:	Outpatient clinic
Implementer variables reported:	Relevant certification, education, training, and employment
Participant characteristics reported:	Age, gender, race, ethnicity, diagnosis, relevant skills and deficits related to liquid consumption
Safety measures reported:	Developmentally appropriate and safe structured seating, initial and ongoing assessment and monitoring from a multidisciplinary team of providers with expertise in pediatric feeding disorders, mouth checks and meal termination criteria

**TABLE 2:** Participant demographics.

Participant	Age (Years)	Diagnosis	Race & Ethnicity	Treatment
Alan	1	Prematurity	White & Non‐Hispanic	EE + NCA
Blake^a^	2	Prematurity	White & Non‐Hispanic	EE + AE + NCA
Cody	3	Developmental delay, prematurity, gastroesophageal reflux, failure to thrive	White & Non‐Hispanic	EE + AE + NCA
Dalia	3	Autism	Black & Non‐Hispanic	EE
Fabio^a^	4	Developmental delay	Hispanic (Mexican)	EE + DR
Levi^a^	3	Developmental delay, prematurity	White & Non‐Hispanic	EE + NCA
Liam	1	N/A	White & Non‐Hispanic	EE + DR
Micah	1	N/A	White & Non‐Hispanic	EE + NCA
Sara	1	Prematurity	White & Non‐Hispanic	EE + DR
Siya	3	Autism, developmental delay, prematurity, gastroesophageal reflux, failure to thrive	Asian Indian	EE + DR
Simon	3	Autism, developmental delay	Black & Non‐Hispanic	EE + DR
Maria	2	Failure to thrive	White & Non‐Hispanic	EE + AE + NCA
Kazi	2	Prematurity	White & Non‐Hispanic	EE + AE + NCA
Ava	1	N/A	Asian Indian	EE + AE + NCA
Cadell^a^	1	Gastroesophageal reflux, Failure to thrive	White & Non‐Hispanic	EE + AE + NCA
Jay^a^	3	N/A	Hispanic	EE + AE + DR
Ella^a^	1	Prematurity	White & Non‐Hispanic	EE + NCA

*Note*: EE = escape extinction; NCA = noncontingent access; AE = attention extinction; DR = differential reinforcement.

^a^ = gastrostomy tube dependence.

Due to the complex etiology of feeding difficulties, a multidisciplinary team of professionals with expertise in pediatric feeding disorders evaluated the children before admission to the clinical program. The multidisciplinary team included a registered dietitian, pediatric gastroenterologist, doctoral‐level licensed psychologist and/or board‐certified behavior analyst, and a speech–language pathologist with expertise in pediatric swallow safety. Safety of oral feeding and readiness for the program were confirmed by the speech–language pathologist before each participant began treatment, and all participants were cleared medically (i.e., no further medical testing recommended, all medical conditions treated and stable).

Before the admission, the registered dietitian used caregiver‐collected 3‐day food records to calculate each child's calorie, protein, fluid, and nutritional needs. Based on this analysis, the registered dietician provided recommendations to caregivers for target foods and liquids that would improve the child's nutrition and support hydration and calorie needs. The registered dietician and feeding program supervisors also asked that caregivers select foods and liquids that were accessible, easily integrated into home meals (e.g., something the family would eat regularly), affordable, and safe for the child to consume (i.e., no allergies or intolerances).

We initially conducted this evaluation to determine the efficacy of a standard clinical procedure (i.e., escape extinction plus re‐presentation; Kaur et al., 2025). The intensive feeding program supervisors sought to better understand whether re‐presentation should be included within a function‐based treatment to treat liquid expulsion. There were two phases of informed consent. First was to obtain caregiver consent for their child to receive treatment involving some combination of escape extinction and reinforcement to address their child's liquid expulsion. Feeding program supervisors met with caregivers to review various treatment pathways for increasing consumption of a nutritional liquid as a part of general clinical care. All treatment pathways that were reviewed with caregivers were evidence based and behavior analytic (e.g., demand fading, blending); however, caregivers ultimately decided whether escape extinction was desirable and preferred relative to others. If caregivers consented to treatment, feeding therapists conducted the comparison (i.e., escape extinction with and without re‐presentation) to determine which treatment was most effective for each child. Caregivers were informed that they could stop or change treatment at any time, but this did not occur throughout the clinical evaluation. Caregivers were encouraged to observe treatment from an adjacent observation booth, and most of the caregivers in the current study were present for most sessions.

After a period of 2 years, the clinical team analyzed the accumulated data to determine whether the program should continue with the standard practice (i.e., embed re‐presentation when escape extinction is conducted). The clinical team then planned to share the program evaluation findings as a prospective consecutive controlled case series, given that all children who were exposed to the treatment comparison met predefined inclusion criteria (described below). Table 1 includes the reporting guideline checklist (Kaur et al., 2025). Therefore, the second phase of consent involved obtaining written and verbal consent from caregivers for their child's deidentified data to be included and shared. All procedures related to compiling and publishing clinically obtained data were approved by the university's institutional review board. Caregivers were informed that they could withdraw their consent at any time for their child's data to be shared; however, this did not occur for any participant in the study.

### Personnel and safety management

The clinical team included feeders, observers (i.e., data collectors), doctoral‐level feeding program supervisors, and the multidisciplinary team. Feeders and observers were individuals who were employed at the feeding program and who had bachelor's, master's, or doctoral degrees in applied behavior analysis, psychology, or a related field (e.g., social work). Feeders and observers also included master's‐ or doctoral‐level trainees (e.g., predoctoral interns) in similar fields, who were completing experiential practicum rotations in the feeding program. The doctoral‐level feeding program supervisors (i.e., licensed psychologists or doctoral‐level board certified behavior analysts) coordinated ongoing collaboration with the multidisciplinary team, facilitated all treatment decisions, maintained consistent contact with participant caregivers, oversaw training of feeders and observers, and were responsible for the child's progression through their day‐treatment admission. These supervisors and additional masters‐level board certified behavior analysts who had worked in the feeding program for at least 5 years taught feeders to implement all procedures using written and vocal instructions, modeling, role play, and feedback. The master's‐level, board‐certified behavior analysts also taught observers to collect data on all primary dependent variables using similar training strategies.

Members of the multidisciplinary team maintained ongoing contact throughout the child's admission and the study. For example, feeders and feeding program supervisors regularly met with the speech–language pathologist to ensure safe seating and posture and appropriate bolus sizes for liquids; the registered dietician to ensure participants were meeting their nutrition, hydration, and calorie needs; and the pediatric gastroenterologist to wean tube feeds and check in on unexpected medical or illness‐related concerns if they arose. Members of the multidisciplinary team also conducted periodic observations of treatment meals to monitor for safety and progress.

The clinical team implemented preventive safety strategies and conducted ongoing monitoring based on recommendations provided by the multidisciplinary team throughout the study. All team members who worked with participants were trained to administer CPR, first aid, and basic life support. During sessions, feeders followed standard clinical safety precautions that included methods for safe deposit and re‐presentation of liquids (i.e., holding the cup to the side of the participant's lips and not depositing or re‐presenting the drink while the participant was coughing, gagging, or vomiting). If the participant tilted their chin or head downward during drink deposits or re‐presentations, the feeder gently placed the open palm of their nonfeeding hand on the child's forehead to lift, support, and assist the child in maintaining an open airway (Redstone & West, 2004). Feeders used a small bolus for each drink presentation and re‐presentation (i.e., 2 or 4 ml). Feeders also maintained a consistent drink presentation rate during all sessions (e.g., one, 2‐ml drink presented every 30 s, maximum of five drink presentations per session), capped sessions at 10 min, conducted mouth checks between each drink presentation, and ensured there was no liquid larger than the size of a pea inside the child's mouth at the end of the five‐trial session. If a multidisciplinary team member had alternative recommendations for sessions (e.g., a change to the rate of drink presentations), the clinical team would have used this structure, but this did not occur.

Feeding program supervisors also taught feeders and observers to identify and listen for atypical vocal sounds (e.g., wetness or raspiness, congestion; Groves‐Wright et al., 2010) that were different from the child's baseline status and occurred or persisted outside of an identified illness because these signs could indicate aspiration or improper management of food or liquid. Observers recorded occurrences of coughs, gags, and emesis (i.e., vomit) during all meals. Feeding program supervisors would have contacted the speech–language pathologist or the child's medical team if concerning vocalizations emerged, but this did not occur with any participant in the study.

### Setting and materials

Participants attended an intensive day‐treatment feeding program within a hospital‐ and university‐based facility. Participants attended appointments daily (i.e., Monday through Friday) from approximately 9 a.m. to 4 p.m. for 8 to 16 weeks, depending on the child's rate of goal attainment. Participants attended the program for 4 to 6 hr per day, depending on their admission type (i.e., half‐ or full‐day admission). Participants had three or five meals daily (for half‐ and full‐day admissions, respectively).

Study sessions were conducted in 4‐ × 4‐m treatment rooms equipped with one‐way observation capabilities and two‐way communication systems. Adult‐sized wooden tables and a variety of chairs for seating were inside each treatment room. Feeders obtained the participants' height and weight measurements at the beginning of the admission and at weekly intervals throughout the study. Feeding program supervisors and multidisciplinary team members used height and weight information to identify and confirm safe seating arrangements for each participant. That is, the clinical team selected a seat that enabled the child to sit upright to maintain an open airway and ensured that the child could be seated in a 90°‐90°‐90° position (i.e., angles between the upper torso, thighs, and lower legs, respectively; Redstone & West, 2004). Chairs included a variety of age or developmentally appropriate options such as highchairs for children who were 19 kg or less, booster seats secured to regular adult‐sized chairs for children who weighed up to 28 kg, regular adult‐sized chairs for children who were 28 kg or greater and could support sustained upright posture, or a Special Tomato Soft‐Touch sitter that was secured to a regular adult‐sized chair for children of any weight who required additional support to maintain upright posture and core stability. The child's height and weight determined the size of the Special Tomato Soft‐Touch sitter (sizes 1 through 5). Feeding program supervisors and multidisciplinary team members selected chairs primarily based on the child's height, weight, and developmental status. When possible, team members selected seating that was age appropriate. However, safe posture was prioritized over a child's age. For example, if a 4‐year‐old child could not maintain upright posture in a booster seat and had a lower weight (e.g., 15 kg), a highchair would have been used. Feeding program supervisors confirmed each caregiver's acceptability and consent for use of structured seating.

If a child did not independently transition to the treatment room or sit in the chair, feeders conducted a variety of interventions to target this variable before addressing meal‐related instructions (e.g., noncontingent access to preferred items during transitions). Feeders and observers also spent time building rapport with participants during the initial assessment period, between meals (e.g., playtime in the playground), and before meals started while seated at the table. Following the study, feeding program supervisors, with guidance from the multidisciplinary team, worked with each participant's caregivers to remove structured seating arrangements as children demonstrated a consistent upright posture without additional support.

Various items were present in the treatment rooms including feeding utensils (e.g., flexi‐cut cups, plastic syringes, plastic snap‐on bibs with spill‐catcher pouches, rubber‐coated baby spoons), food trays, a digital food scale, timers, and additional treatment‐specific stimuli (e.g., preferred toys). Treatment rooms also included materials to maintain hygiene and cleanliness (e.g., nonlatex gloves, sanitizing wipes, paper towels), the child's target liquid, one or two laptop computers for data collection, and adult‐sized chairs for feeders and observers.

### Dependent variables, response measurement, procedural fidelity, and interobserver agreement

Observers collected frequency and duration data on laptop computers equipped with DataPal 1.0 (i.e., a beta version of BDataPro software; Bullock et al., 2017). Observers collected data from inside the treatment room, which allowed them to see the contents of the participant's mouth or whether liquid had been expelled. The primary dependent measures were mouth clean and expel (per opportunity). Observers also collected data on child secondary measures such as active and nonactive liquid acceptance, IMB, and negative vocalizations. Finally, observers collected data on procedural fidelity measures (described below).

*Active acceptance* was defined as the child opening their mouth within 5 s of the initial drink presentation without negative vocalizations, which initiated the feeder's deposit of the entire bolus of liquid into the mouth (additional procedural details below). If negative vocalizations occurred (i.e., the child cried, whined, or made negative statements, regardless of the volume), acceptance was only scored if the child opened their mouth *and* moved toward the cup, which initiated the deposit response within 5 s of the drink presentation. *Nonactive acceptance* was scored when the feeder deposited the entire bolus of liquid into the mouth after 5 s. Observers scored *mouth clean* 15 or 30 s after the liquid entered the mouth if no liquid, pea‐sized or greater, remained inside the mouth (not due to expulsion or vomiting). Of note, the opportunity for mouth clean depended on whether acceptance occurred. Observers did not score mouth clean if acceptance did not occur because there was not an opportunity for mouth clean if liquid did not enter the mouth (see Engler et al., 2023). Thus, in the current study, when observers scored a zero for mouth clean in baseline, either there was no opportunity or acceptance occurred but mouth clean did not occur.

Observers scored *expel* when liquid, pea‐sized or greater, exited the mouth and was no longer on the tongue or on the inner portion of the lips. Therefore, observers could only score expel when liquid pea‐sized or greater had entered the mouth. A *re‐presentation* was scored when the feeder replaced the approximate amount of expelled liquid back into the child's mouth within 3 s of an expel. Observers also scored re‐presentation if the feeder had to retrieve a fresh drink to replace into the child's mouth after expel if the original bolus was contaminated (e.g., expelled onto an unsanitary surface; mixed with saliva or mucous).

Because the rate of expel may be higher during re‐presentation conditions (additional opportunities to expel), we converted expels into responses per opportunity. In each session during all conditions, there were up to five opportunities for liquid to be inside the child's mouth (i.e., 5‐trial sessions). In each trial, the child could engage in active acceptance, nonactive acceptance, or no acceptance. During the re‐presentation condition, there were additional opportunities for liquid to be inside the mouth (i.e., each time the feeder redeposited expelled liquid). Thus, *expel per opportunity* in the re‐presentation condition was calculated by summing the total number of expels and dividing that sum by the number of acceptances (active and nonactive) plus the number of re‐presentations. Expel per opportunity in the no re‐presentation condition was calculated by summing the total number of expels and dividing that sum by the number of acceptances (active and nonactive).

Observers scored occurrences of *IMB* each time the participant (a) touched or hit the cup, the liquid, or the feeder's hand or arm from the elbow down during a presentation or re‐presentation; (b) blocked their mouth with their hand, bib, or a toy; (c) threw the cup; and (d) moved the middle of their mouth approximately 45 degrees or 6.3 cm in any direction except toward the cup, changed direction, or paused for 1 s or more and moved their mouth another 45 degrees or 6.3 cm in any direction except toward the cup. The total frequency of IMB was converted into *responses per minute* by dividing the total frequency of IMB by the duration that the cup was within arm's reach of the participant during the session.

A second observer independently collected data for a mean of 27% of sessions (range: 6%–52%) across participants. DataPal 1.0 separated the duration of each session into 10‐s intervals. Interobserver agreement for mouth clean and expel was calculated by dividing the number of agreements (both observers scored an occurrence or both observers did not score an occurrence across 10‐s intervals) by the number of agreements plus disagreements (i.e., one observer did and the other did not score an occurrence of mouth clean or expel) and converting this ratio to a percentage. Mean interobserver agreement was 97.75% (range: 72.24%–100%) for mouth clean and 97.23% (range: 68.37%–100%) for expel.

At least one observer collected data on correct utensil placement during 100% of sessions for all participants. This was scored when the feeder maintained the cup to deposit or re‐present liquid in the correct position (see below under General study procedures and Treatment sections). Observers depressed a key during the initial drink presentation if the feeder presented the cup in the correct position and depressed the key to stop measuring correct utensil placement after three consecutive seconds of incorrect utensil placement. For example, if the feeder did not re‐present the cup within 3 s of expel, the observer would depress the duration key because the cup placement was incorrect during re‐presentation. In this scenario, the observer would not restart the correct‐utensil‐placement key until the feeder had resumed correct placement. As another example, if the feeder did re‐present the cup to the lips following expel during the *no re‐presentation* treatment condition, the correct utensil placement key would have been turned off. Procedural fidelity was calculated by dividing the duration of correct utensil placement by the total session time. Mean correct utensil placement was 94.99% across participants (range: 22.31%–100%). Correct utensil placement was mostly high across participants as indicated by the mean (i.e., 94.99%); however, if correct utensil placement fell below 80% for three consecutive sessions, those feeders would complete re‐training with feeding program supervisors (e.g., role play with feedback).

### Experimental design

The clinical team used a combined reversal and multielement design for each participant's clinical evaluation as a part of a prospective consecutive controlled case series (Hagopian, 2020). Specifically, we used a reversal design for all participants except Mary, Cody, Fabio, and Ella, to demonstrate experimental control of the escape‐extinction‐based treatment (i.e., nonremoval of the cup). We conducted a multielement comparison to assess levels of mouth clean and expel per opportunity in re‐presentation and no re‐presentation conditions. Feeders randomized and counterbalanced the order of the conditions of the multielement comparison throughout the study. We used the framework of a consecutive controlled case series to consecutively enroll participants who all engaged in liquid expulsion and met the inclusion criteria. Therefore, the consecutive controlled case series was used to evaluate the generality of a specific procedure that was employed across (e.g., re‐presentation) multiple cases with a common characteristic (e.g., liquid expulsion).

### Preassessment

#### Parent intake questionnaire

Feeding program supervisors sent an intake packet to caregivers before participants' admission to the day‐treatment program and enrollment into the study. The intake packet included questions about the child's medical and feeding history, previous feeding services, current feeding routines and meal schedule, oral‐motor patterns that could represent skill deficits (e.g., chronic open‐mouth posture, saliva pooling), sleep and toileting routines, and a 3‐day food record. The study and multidisciplinary team members reviewed the questionnaires to prepare safe seating and other safety precautions (e.g., bolus size for liquids, type of cup) and to ensure the child was appropriate for an intensive day‐treatment admission. The parent intake questionnaire is available on request from the first author.

#### Initial liquids observation

At the beginning of each child's admission to the day‐treatment program, the clinical team conducted a home baseline observation and a structured meal observation involving various liquid bolus sizes across self‐ and non‐self‐feeder arrangements (Peterson et al., 2018). The home baseline and structured mealtime observations were implemented by one or several of the child's primary caregivers.

During the home baseline observation, feeders asked caregivers to present both preferred and nonpreferred solids and liquids in separate conditions. Observers collected data on child (e.g., refusal, acceptance) and caregiver (e.g., coaxing, removal of the drink) behavior. The clinical team conducted the home baseline meal observation to better understand child and caregiver behavior during meals that were conducted in a manner similar to that in the home setting.

During the structured mealtime observation, feeders asked caregivers to present 2 ml of the target liquid every 30 s and respond to their child how they typically would at home. The clinical team conducted the structured observations to gather baseline information regarding (a) the child's acceptance of liquids when given the opportunity to self‐feed versus being fed by a caregiver, (b) the child's oral‐motor skills with cup drinking (e.g., liquid running from the mouth, improper lip seal on the edge of the cup), (c) the types of caregiver responses that occurred following appropriate and inappropriate child behavior, and (d) the child's behavior when antecedent‐based manipulations were made (e.g., decreased bolus, presenting drinks at appropriately timed intervals). Based on these observations, multidisciplinary team recommendations, and caregiver goals, the clinical team decided to initiate a treatment evaluation for liquids using a non‐self‐feeder format for all 17 participants.

#### Preference assessment

Feeders conducted preference assessments with participants to identify tangible stimuli that could be used during treatment. The clinical team programmed differential or noncontingent reinforcement with tangible stimuli for six and 11 participants, respectively. These reinforcement‐based components were in place to increase acceptance of liquids or were selected by caregivers as a method to improve the overall mealtime experience for the child and not implemented specifically to reduce expulsion. One participant (Dalia) did not have reinforcement‐based treatment components due to caregiver preference. Feeders conducted paired‐stimulus, multiple‐stimulus‐without‐replacement, or free‐operant preference assessments based on the child's age and cognitive level (DeLeon & Iwata, 1996; Fisher et al., 1992; Roane et al., 1998).

#### Functional analysis

The clinical team conducted a functional analysis of IMB to identify treatment for participants and used similar procedures to Kirkwood et al. (2021). Across participants, only escape and attention test conditions, or both, were evaluated and compared with a control condition. The clinical team determined through visual inspection of the data that IMB was maintained by escape only for Mary, Alan, Dalia, Fabio, Levi, Liam, Micah, Sara, Siya, Simon, and Ella and determined that IMB was multiply maintained by escape and attention for Blake, Cody, Maria, Kazi, Ava, Cadell, and Jay.

### General procedures

Participants experienced multiple 30–45‐min therapy meals each day with 40‐ or 45‐min breaks between meals. Each meal consisted of multiple five‐drink sessions. Not all meals involved liquids (i.e., some meals each day included solids treatment). However, given the focus of the current study, only liquids‐based treatment evaluations are included. Five‐drink sessions never lasted longer than 10 min. Participants experienced brief breaks between each session while feeders or observers weighed liquids and prepared data collection to begin another session. The number of sessions per meal depended on the scheduled meal duration (e.g., 30 or 45 min) and the duration of the sessions. The number of five‐drink sessions that were conducted per meal ranged from two to seven sessions across participants. Feeders presented 2 ml of liquid in the cup across all conditions for all participants, except for Fabio. For Fabio, feeders presented 4 ml of liquid across all conditions because this was recommended by members of the multidisciplinary team. The clinical team collaborated with the participant's physician and caregiver or the multidisciplinary team members to adjust the participant's tube‐ or oral‐feeding schedule so that the participant did not have a meal for at least 30 min before therapy meals. We asked caregivers to track intake (i.e., tube and oral feedings) that occurred outside of therapy meals and recommended that they implement consistent meal schedules throughout the study.

During breaks between therapy meals, participants had access to leisure items and to various areas of the clinic (e.g., outdoor and indoor large playgrounds, toy rooms). No meal‐ or non‐meal‐related tasks were scheduled during breaks between meals. Participants attending full‐day admissions had one long break (e.g., 90–120 min) in the afternoon for naps (if needed, based on caregiver request).

Across all conditions of the study, feeders initiated a drink presentation by bringing the cup to the child's lips with a vocal instruction (i.e., “Take a drink”). If the child opened their mouth to accept the drink, the therapist touched the opening of the cup to the center of the child's bottom lip while slowly tilting the cup upward to deposit the liquid inside the mouth. If the child accepted the drink within 5 s of the presentation, therapists provided brief, behavior‐specific praise (e.g., “Great job taking your drink so fast!”). The feeder conducted mouth checks 15 or 30 s after drink acceptance by vocally instructing the child to open their mouth while providing a model (i.e., “Show me, ahh”). If the participant had a mouth clean, the feeder provided behavior‐specific, enthusiastic praise (e.g., “Great job swallowing your drink”). If participants did not have a mouth clean (due to expel or pack), the feeder reminded them to, “Finish swallowing your drink.” If participants clenched their lips or teeth during mouth checks, therapists reissued the vocal instruction one additional time and gently touched a rubber‐coated baby spoon to the corner of the participant's lips to provide an additional prompt. The feeder terminated vocal instructions for mouth checks after 1 min and proceeded to the next drink presentation if the child did not show, but this rarely occurred throughout the study.

#### Baseline

During baseline sessions, the same general study procedures were in place (e.g., feeder presented the cup to the child's lips and gave the instruction, “Take a drink,” completed mouth checks, and delivered praise for active acceptance and mouth clean). In addition, feeders included specific consequences following occurrences of IMB based on the results of the functional analysis. If the functional analysis results indicated both escape and attention functions (i.e., for Blake, Cody, Maria, Kazi, Ava, Cadell, and Jay), the feeder delivered 30 s of escape from the drink presentation (removal of the cup) and attention that was matched to the type of attention caregivers delivered during initial meal observations contingent on IMB (e.g., coaxing). If functional analysis results indicated an escape function only (i.e., for Mary, Alan, Dalia, Fabio, Levi, Liam, Micah, Sara, Siya, Simon, and Ella), the feeder delivered 30 s of escape from the drink presentation (no attention) contingent on IMB. For six participants (Fabio, Liam, Sara, Siya, Simon, and Jay), baseline also included differential reinforcement for acceptance. That is, if the participant engaged in active acceptance, a preferred item identified from the preference assessment was delivered for 30 s. Across all baseline sessions, if passive refusal occurred (e.g., no acceptance and the absence of IMB), the feeder would keep the cup stationary in the same position in space where they had initially presented the drink for the remainder of the 15‐ or 30‐s trial. At the end of the trial, the feeder removed the cup and presented the next trial.

#### Treatment

##### Reinforcement and other components common to both treatments

As noted, for all participants praise was provided for active acceptance and mouth clean. As a part of our regular clinical practice, the clinical team gave caregivers a choice of including other additional treatment components that included noncontingent access, differential reinforcement, a combination, or none of these. The clinical team explained each of these components before asking caregivers to select which ones they preferred (if any). Based on caregiver selection, the feeder included noncontingent access to preferred items and attention for Mary, Alan, Blake, Cody, Levi, Micah, Maria, Kazi, Ava, Cadell, Jay, and Ella. During noncontingent access, the feeder delivered continuous access to a preferred toy identified in the preference assessment and attention (in the form of talking, singing, and playing) throughout the entire session. If a participant's treatment included attention extinction and noncontingent access, the feeder did not provide caregiver‐matched attention following occurrences of IMB and instead delivered continuous, positive interactions with tangible items noncontingently. Differential reinforcement was included for Fabio, Liam, Sara, Siya, Simon, and Jay. During differential reinforcement, feeders delivered attention along with one of the child's highly preferred items contingent on active acceptance for 30 s. Feeders did not remove attention or tangible items if expel occurred after acceptance. Dalia's caregivers were the only caregivers who chose to include no additional treatment components (no noncontingent access or differential reinforcement); however, she continued to receive praise for active acceptance and mouth clean.

##### Function‐based treatment with re‐presentation

Treatments were matched to identified escape and attention functions. During function‐based treatment with re‐presentation, feeders used the same procedures as were used in baseline and included additional reinforcement when applicable (described above). Attention extinction was included for Blake, Cody, Maria, Kazi, Ava, Cadell, and Jay. During attention extinction, feeders no longer provided attention following occurrences of IMB. Feeders implemented escape extinction with children who had identified escape functions (all participants). During escape extinction, the feeder kept the cup touching the participant's lips until the participant opened their mouth and the therapist deposited the drink inside the mouth or until the session time cap was met (escape extinction). The feeder held the cup at the corner of the participant's lips if the participant was coughing, gagging, or vomiting during the initial presentations or during re‐presentations to ensure nothing entered the participant's mouth that could obstruct the airway. The feeder additionally held the cup to the corner of the participant's lips and did not deposit the liquid if the participant's head was not upright. If the participant engaged in crying that posed safety risks (e.g., sharp inhalations or gasps) the feeder paused the drink deposit. When the child was no longer gagging, coughing, or vomiting, the feeder resumed the general procedure. If vomiting occurred, feeders did not resume drink presentations until the child and the feeding area were clean and cleared of any contaminated materials (e.g., emesis) and after the feeder had retrieved fresh utensils (stored in the same treatment room). Feeders never re‐presented emesis or contaminated liquid. For example, if liquid was expelled onto the floor or contacted emesis, the feeder retrieved a fresh drink. Similarly, if liquid ever mixed with excessive salvia or mucous, the feeder gently wiped it away and retrieved a fresh bolus of liquid into the cup.

The feeder re‐presented expelled liquid by scooping the expelled liquid with the cup or obtaining a fresh bolus of liquid that approximated the amount of liquid that was expelled. The feeder used the same drink presentation procedures to re‐present expelled liquid as described above. The cup remained at the participant's lips until the participant opened their mouth again, at which point the feeder deposited the liquid into the mouth. If the feeder needed to move the cup away from the lips to retrieve the expelled liquid, the feeder moved as quickly as possible to minimize the delay to re‐presentation (e.g., re‐presented liquid within 3 s of expel). When possible, the feeder would keep the cup tilted at the front of the child's lips while depositing the approximate amount of expelled liquid into the cup with a plastic syringe. Function‐based treatment sessions never lasted longer than 10 min.

##### Function‐based treatment without re‐presentation

Feeders used the same treatment procedures described as the function‐based treatment with re‐presentation condition; however, re‐presentation was not used. Thus, the drink was not presented again if expels occurred. Regardless of whether expel occurred, feeders transitioned to the next drink presentation and provided no attention and no other differential consequences for expel at the end of each 15‐ or 30‐s interval.

## RESULTS

Feeding program supervisors used visual inspection to make clinical decisions throughout treatment and determine when to move to the next phase of treatment. The authors of this article identified patterns of responding post hoc based on visual inspection of the figures and comparisons of averages across conditions for mouth clean and expel. After the comparison, feeders continued with one treatment (function‐based treatment with or without re‐presentation) based on whichever condition led to highest levels of mouth clean and lowest levels of expel. If both treatments produced similar outcomes, caregivers selected the final treatment. Tables 3 and 4 display mean percentages of mouth clean and mean expels per opportunity across participants and conditions.

**TABLE 3:** Mean percentage of mouth clean across conditions.

Participant	Baseline 1	Re‐presentation 1	No re‐presentation 1	Baseline 2	Re‐presentation 2	No re‐presentation 2
Alan	0	97.33	4	11.66	100	3.48
Blake	0	84.91	0.38	0	87.22	0
Cody	0	100	44.11	71.73	99.66	N/A
Dalia	0	81.94	50.97	16.67	95	62.86
Fabio	0	29.17	4.80	64.17	91.29	N/A
Levi	0	96.74	52.12	65.86	94.91	84.65
Liam	0	82.76	1.55	0.95	75.71	1.43
Micah	0	94.26	50.41	25.38	100	92.50
Sara	0	95.57	19.09	20	99.52	51.61
Siya	0	92.67	0.56	0	100	0
Simon	10.77	80	68	63.18	72.50	66.67
Maria	0	97	88.75	78.24	100	100
Kazi	0	2.69	0	18.82	50.29	44.71
Ava	0	97.06	91.18	12.31	100	96.25
Cadell	55.38	97.14	100	93.83	100	93.61
Jay	0	86.90	87.41	68.24	73.33	98.57
Ella	0	16.94	6.43	N/A	N/A	N/A

*Note*: N/A = not applicable.

**TABLE 4:** Mean expel per opportunity across conditions.

Participant	Baseline 1	Re‐presentation 1	No re‐presentation 1	Baseline 2	re‐presentation 2	No re‐presentation 2
Alan	0	0.40	0.97	0.78	0.48	1.01
Blake	0	0.59	1.38	1.12	0.62	1.09
Cody	0	0.37	0.67	0.11	0.03	N/A
Dalia	0	0.29	0.58	0.04	0.10	0.46
Fabio	0	0.98	2.38	0.47	0.16	N/A
Levi	0	0.34	0.54	0.17	0.11	0.17
Liam	0	0.74	1.20	1.02	0.69	0.96
Micah	0	0.37	0.59	0.09	0.08	0.14
Sara	0	0.53	0.83	0.25	0.34	0.47
Siya	0	0.65	1.05	1	0.67	1
Simon	0	0.17	0.14	0.19	0.05	0.03
Maria	0	0.13	0.16	0.01	0.08	0.03
Kazi	0	0.36	0.63	0.04	0.06	0.09
Ava	0	0.01	0.08	0	0.01	0.01
Cadell	0	0.09	0.03	0.03	0.01	0.05
Jay	0	0.05	0.10	0.44	0.37	0.03
Ella	0	0.83	0.99	N/A	N/A	N/A

*Note*: N/A = not applicable.

Across the 17 participants, five general patterns of responding emerged. Based on visual analysis of the data, re‐presentation resulted in higher levels of mouth clean and lower rates of expel than no re‐presentation for 10 participants (i.e., Alan, Blake, Cody, Dalia, Fabio, Levi, Liam, Micah, Sara, and Siya). For three participants (Simon, Maria, and Kazi), data were variable across re‐presentation and no re‐presentation conditions; however, mouth clean increased and expel decreased in both conditions relative to baseline. For two participants (Ava and Cadell), re‐presentation and no re‐presentation resulted in equal improvement for mouth clean and expel relative to baseline, with less variability between the two conditions. For one participant (Jay), no re‐presentation resulted in higher levels of mouth clean and lower rates of expel than the re‐presentation condition. For one participant (Ella), additional treatment components were needed (i.e., escape extinction with and without re‐presentation was insufficient to increase mouth clean and reduce expel to clinically acceptable levels and rates). For some participants (e.g., Alan), there was little to no overlap between data paths of the re‐presentation and no re‐presentation conditions. However, for other participants (e.g., Dalia), there was greater overlap between conditions, although re‐presentation was ultimately more effective.

**FIGURE 1:**
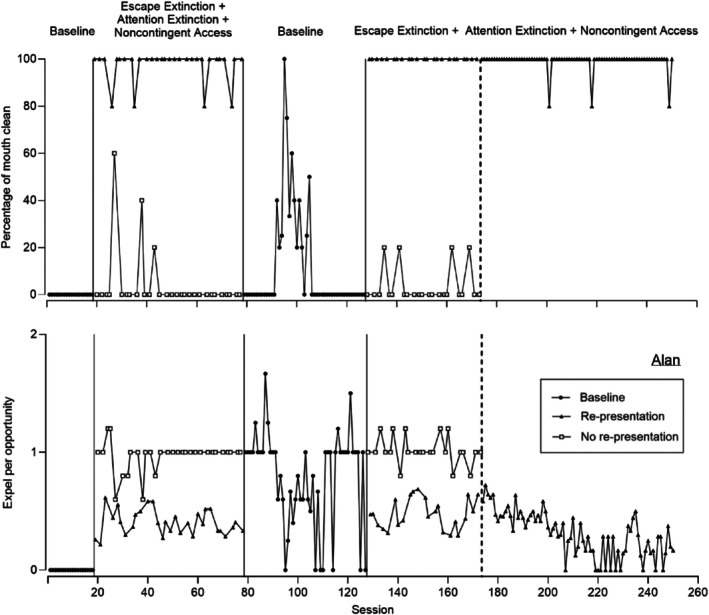
Percentage of mouth clean (top) and expel per opportunity (bottom) for Alan.

**FIGURE 2:**
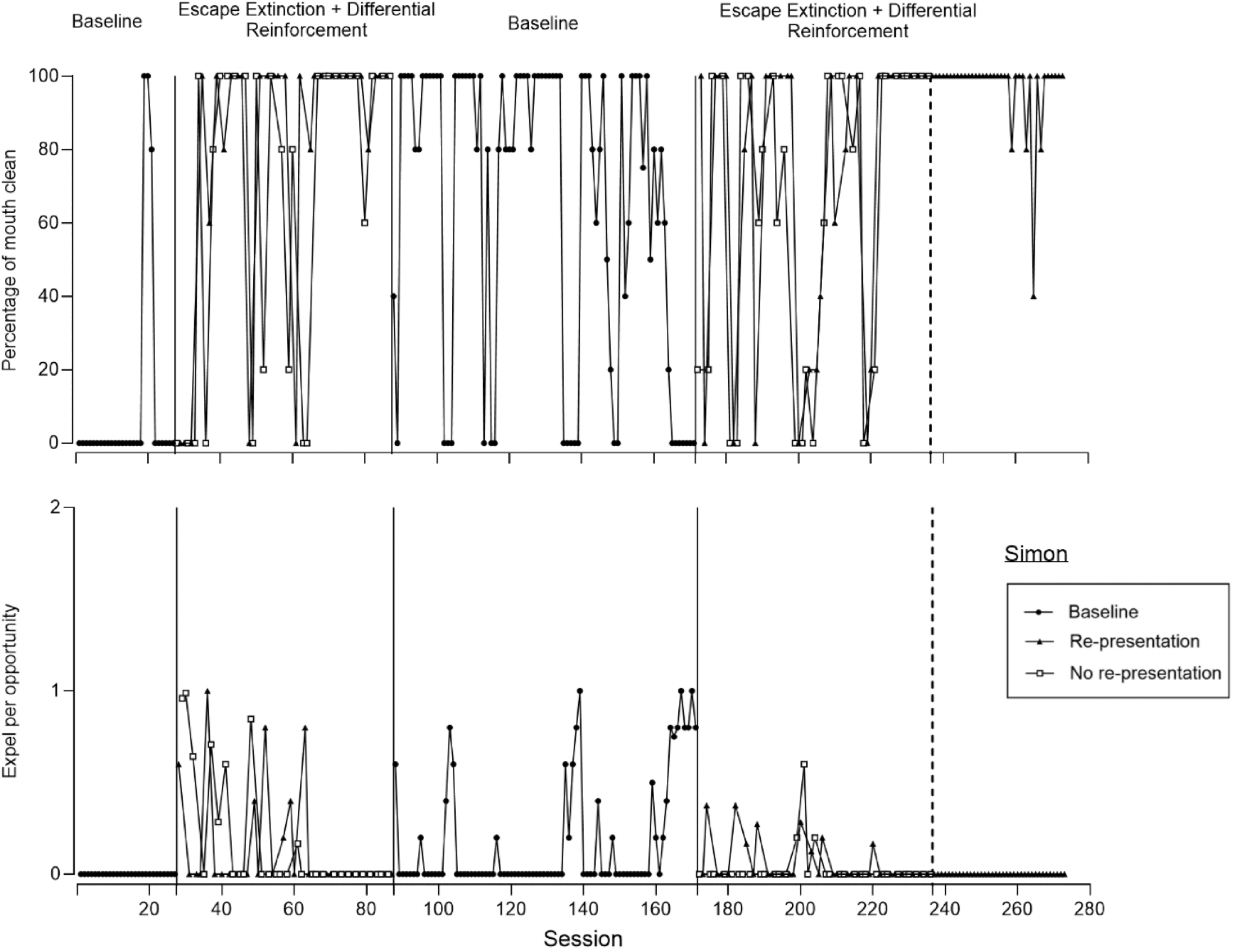
Percentage of mouth clean (top) and expel per opportunity (bottom) for Simon.

**FIGURE 3:**
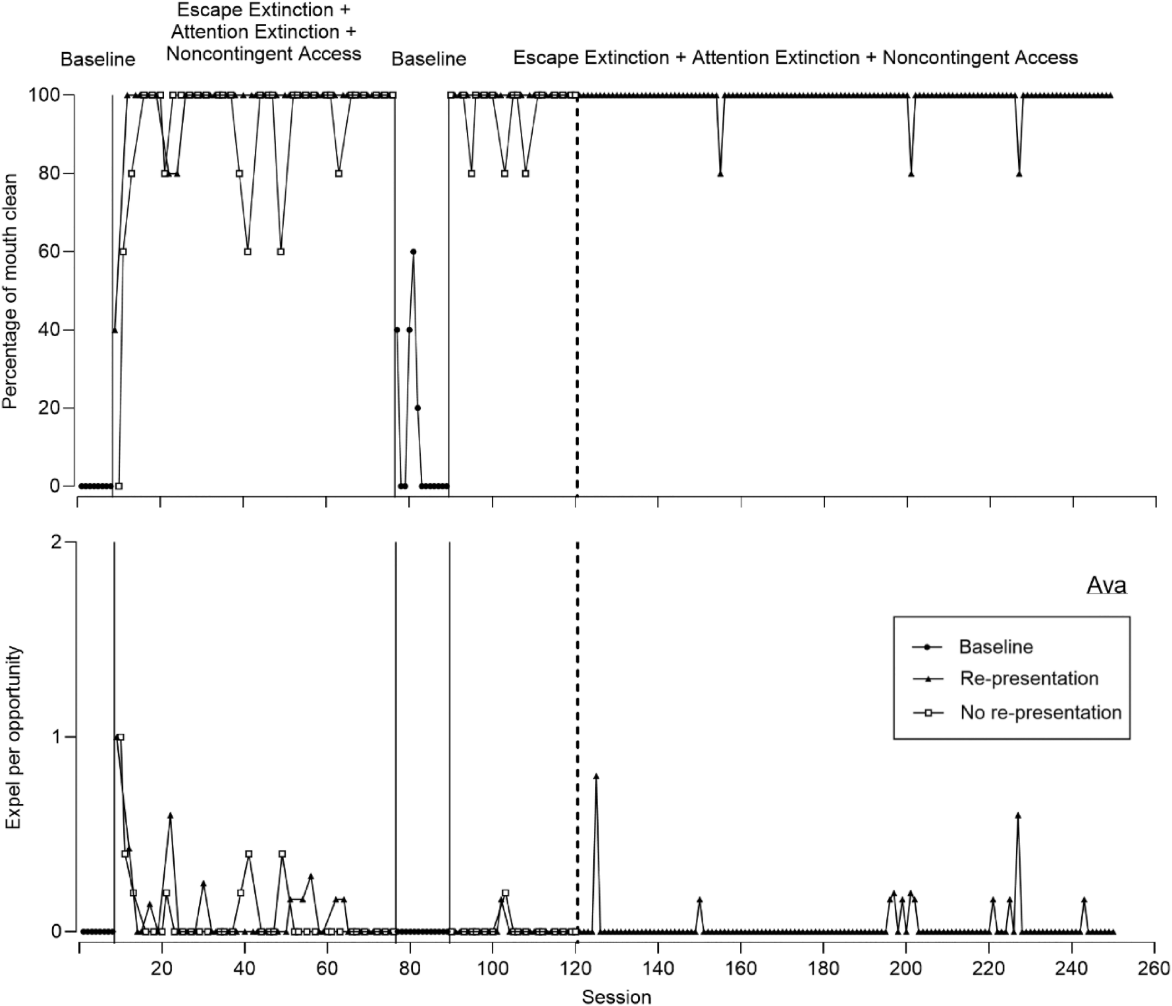
Percentage of mouth clean (top) and expel per opportunity (bottom) for Ava.

**FIGURE 4:**
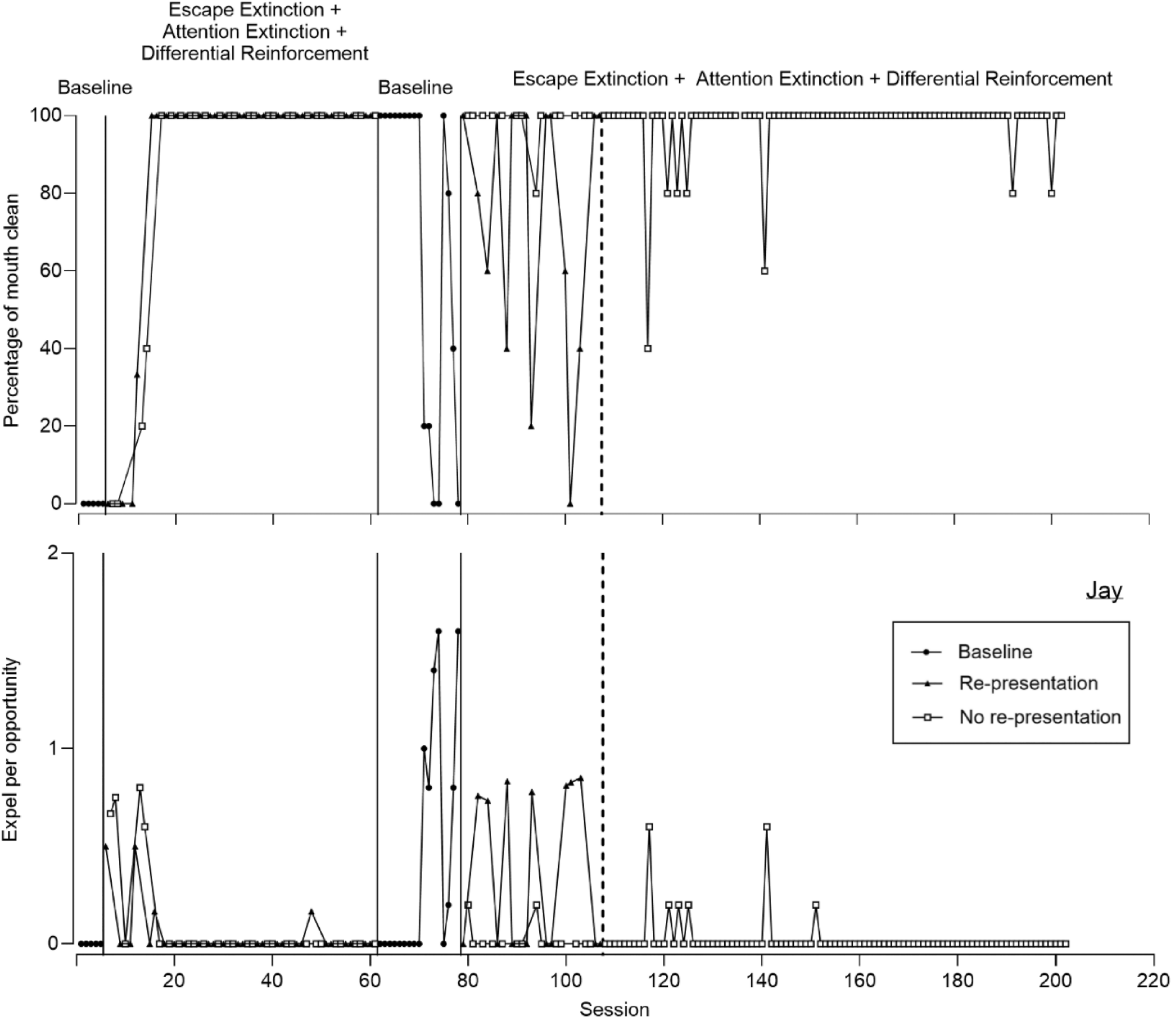
Percentage of mouth clean (top) and expel per opportunity (bottom) for Jay.

**FIGURE 5:**
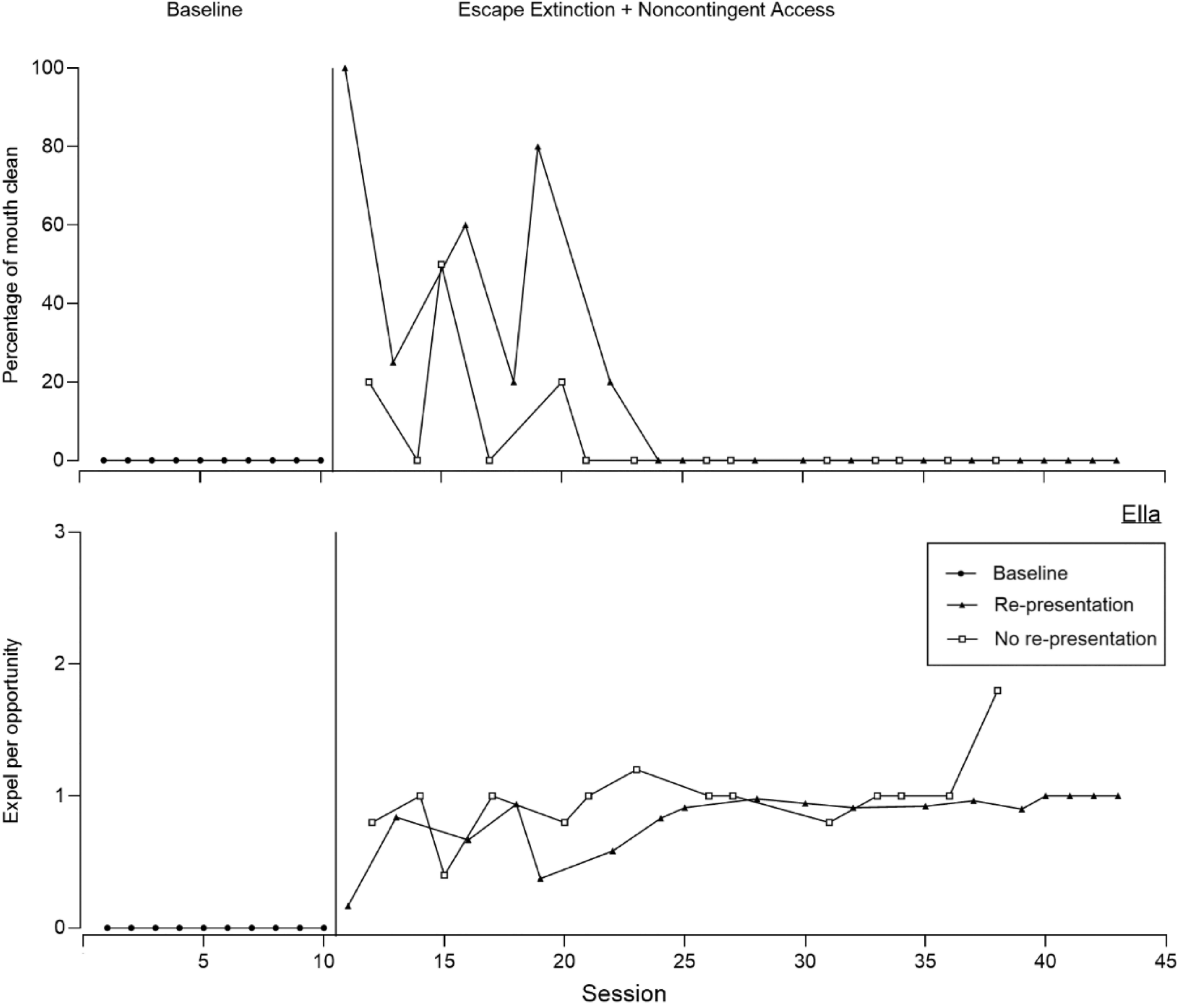
Percentage of mouth clean (top) and expel per opportunity (bottom) for Ella.

Figures 1, 2, 3, 4, 5 display five representative data sets to provide examples of these different outcomes and general patterns that were observed across the 17 participants. Supporting Information B–M include data sets for the remaining 12 participants. Figure 1 displays representative data for the participants for whom re‐presentation was more efficacious than no re‐presentation (i.e., Alan, Blake, Cody, Dalia, Fabio, Levi, Liam, Micah, Sara, and Siya). Figure 1 shows mouth clean (top panel) and expel (bottom panel) for Alan from the treatment comparison. For Alan, acceptance did not occur in the initial baseline; thus, mouth clean (top panel) was zero (*M* = 0%). During the first treatment comparison, mouth clean increased during the condition with re‐presentation (*M* = 97.33%, range: 80%–100%) and was low during the condition without re‐presentation (*M* = 4%, range: 0%–60%). Expel (bottom panel) was low in baseline (*M* = 0). In the treatment comparison, expel increased during re‐presentation (*M* = 0.40, range: 0.22–0.62) and during no re‐presentation (*M* = 0.97, range: 0.60–1.20) but remained higher during the condition with no re‐presentation.

Figure 2 displays representative data for participants for whom there was overlap between re‐presentation and no re‐presentation, with variability across both conditions (i.e., Simon, Maria, and Kazi). Figure 2 displays mouth clean (top panel) and expel (bottom panel) for Simon. Mouth clean (top panel) was variable during baseline (Simon accepted some of the drinks) but, overall, occurred at low levels (*M* = 10.77%; range: 0%–100%). Feeders then introduced the treatment comparison. During the first re‐presentation condition, mouth clean was variable but increased to high, stable levels (*M* = 80%, range: 0%–100%). Similarly, during the first no re‐presentation condition, mouth clean increased variably across sessions but was slightly lower than it was during the re‐presentation condition (*M* = 68%, range: 0%–100%). Expel (bottom panel) was zero during baseline (*M* = 0). When feeders conducted the treatment comparison, expel was low during re‐presentation (*M* = 0.17, range: 0–0.99) and no re‐presentation (*M* = 0.14, range: 0%–1).

Figure 3 displays representative data for participants for whom both re‐presentation and no re‐presentation were effective, with little to no variability (i.e., Ava and Cadell). Figure 3 displays mouth clean (top panel) and expel (bottom panel) for Ava. For Ava, acceptance did not occur in baseline; thus, mouth clean (top panel) was zero (*M* = 0%). During the first comparison phase, mouth clean increased during re‐presentation (*M* = 97.06%, range: 40%–100%) and no re‐presentation (*M* = 91.18%, range: 0%–100%). Expel (bottom panel) also did not occur in baseline (*M* = 0). When the clinical team initiated the treatment comparison, expel was similar across re‐presentation (*M* = 0.10, range: 0–1) and no re‐presentation (*M =* 0.08, range: 0–1).

Figure 4 displays representative data for Jay, who was the only participant with this pattern of responding. Unlike any other participant in the study, Jay's first and second treatment comparison phases differed. In the first comparison phase, re‐presentation and no re‐presentation appeared to be equally effective. In the second comparison phase, treatment without re‐presentation appeared more effective than treatment with re‐presentation. Figure 4 displays mouth clean (top panel) and expel (bottom panel) for Jay. Acceptance did not occur in baseline; thus, mouth clean (top panel) was zero (*M* = 0%). During the first treatment comparison, mouth clean increased to high, stable levels during re‐presentation (*M* = 86.90%, range: 0%–100%) and no re‐presentation (*M* = 87.41%, range: 0%–100%). Similarly, during the first comparison, expel (bottom panel) was low during re‐presentation (*M* = 0.05, range: 0–0.50) and no re‐presentation (*M* = 0.10, range: 0–0.80). Interestingly, mouth clean decreased in the second treatment comparison during re‐presentation (*M =* 73.33%, range: 0%–100%) relative to no re‐presentation (*M* = 98.57%, range: 80%–100%). Expel also increased in the second treatment comparison during re‐presentation (*M* = 0.37, range: 0–0.85) relative to no re‐presentation (*M* = 0.03, range: 0–0.20).

Figure 5 displays representative data for Ella, who was the only participant with this pattern of responding. Figure 5 displays mouth clean (top panel) and expel (bottom panel) for Ella. Mouth clean (top panel) was zero during baseline (*M* = 0%) because Ella never accepted the drink. During the first comparison phase, levels of mouth clean were variable for both re‐presentation (*M* = 16.94%, range: 0%–100%) and no re‐presentation (*M* = 6.43%, range: 0%–50%). Expel (bottom panel) remained elevated across both re‐presentation (*M* = 0.83, range: 0.17–1) and no re‐presentation (*M* = 0.99, range: 0.40–1.80). For Ella, neither condition was sufficient to increase mouth clean or reduce expel. Thus, the clinical team continued to evaluate other treatment components to increase mouth clean and decrease expel. Therefore, there were no second baseline or comparison data for Ella because the comparison was discontinued. Those additional components are not described in detail here because they are beyond the scope of the current study, but the data are available on request from the first author.

## DISCUSSION

In the current study, we conducted a program evaluation project to compare the efficacy of a function‐based treatment with and without re‐presentation for the treatment of liquid refusal. Thus, we carried out a prospective consecutive controlled case series study with 17 children who were admitted to our intensive feeding program. The comparison demonstrated that in the context of function‐based treatment for liquid refusal, re‐presentation was (a) more efficacious than no re‐presentation for 10 of the 17 participants, (b) equally effective as treatment without re‐presentation for five participants, (c) less effective than treatment without re‐presentation for one participant, and (d) equally ineffective across treatments with and without re‐presentation for one participant. Results of the current study are in line with previous research findings demonstrating that re‐presentation is effective as a treatment component for liquid expulsion (Ibañez, Peters, & Vollmer, 2021). The results of this analysis suggest that for most cases in this sample, re‐presentation was a necessary component of a function‐based treatment for liquid refusal. Additionally, through use of a prospective consecutive controlled case series, our clinical team examined the general efficacy of our standard clinical procedures to further improve and refine clinical practice. Clinicians should consider using similar study designs to make and evaluate clinical program improvements.

For 5 of 17 participants, there was no consistently clear differentiation in expel and mouth clean regardless of whether re‐presentation was in place (e.g., Maria). The lack of differentiation across conditions for these participants raises questions regarding whether mouth clean and expel would have improved with nonremoval of the cup alone. For practitioners who do not want to use re‐presentation for concerns relating to possible side effects (e.g., increased emotional responding or IMB), these data suggest that it may not be necessary for all children. However, results should be interpreted with caution, as differences across conditions could have been influenced by carryover effects due to the multielement comparisons. Many stimuli were similar across treatments (e.g., procedures for initial drink presentations were identical to those in re‐presentations); thus, it may not have been discriminable when escape for expel was available.

Re‐presentation was not effective for two participants in the current study. Jay's expulsion continued despite the addition of re‐presentation, although levels of expel were similar in the first‐phase comparison. Jay did not have any known skill deficits; however, Jay did have an identified attention function for IMB. We wondered whether the additional attention provided by the feeder during re‐presentation (i.e., responding to expel by bringing the cup back to the participant's lips) may have functioned as reinforcement, but expel was never assessed in the functional analysis. Ella never reached clinically acceptable levels of mouth clean and expel in either condition and required alternative treatment components. Ultimately for Ella, mouth clean increased and expel decreased with a treatment consisting of thickened liquid, a flipped‐spoon presentation, and a chin prompt, based on recommendations from the multidisciplinary team. Jay and Ella's results suggest that expulsion is a complex behavior that may be affected by environmental contingencies, skill deficits, or both.

The components of re‐presentation are similar to the steps the feeder takes when initially presenting the drink (i.e., bringing the cup to the lips and depositing liquid inside the mouth). However, ubiquitously including re‐presentation could result in unnecessary treatment components or steps, adding to treatment complexity. Because re‐presentation is a form of escape extinction, it could lead to increases in IMB. Although not a primary dependent variable, observers in the current study also measured IMB (Supporting Information N). The mean rate of IMB was similar across conditions, generally lower in both treatment conditions relative to baseline, and reduced for all participants to low levels by the end of the comparison. Thus, our findings did not indicate that re‐presentation led to increases in IMB in the current study. Of note, it may be a difficult for the feeder to maintain procedural fidelity during re‐presentation if there are high rates of IMB that co‐occur with high rates of expel, but this could be evaluated in future research.

To better understand the conditions under which re‐presentation is warranted, researchers should continue to develop and evaluate assessment tools to inform treatment. The authors are not aware of any studies that have assessed expel during functional analyses of IMB. This is not surprising, given that at the time of the functional analysis, children were rarely accepting bites or drinks; thus, expel may not occur. Identification of function could help point therapists toward function‐based interventions. Alternatively, tools to assess a child's oral‐motor status or the conditions under which the child reliably retains liquids could be beneficial (e.g., Scotchie & Borrero, 2023).

For some participants in the current study, expel continued at low to moderate levels across both conditions or required multiple sessions of treatment before we observed meaningful reductions. For Alan, expel persisted at low levels (*M* = 0.48) during the more effective treatment condition (re‐presentation) yet mouth clean was high (*M* = 100%) because he had additional opportunities to swallow the drink. Thus, re‐presentation effectively increased consumption even though expel persisted. Expel can be problematic if it results in reduced consumption. However, it may not always be necessary to target a reduction of expel to zero levels if the child ultimately consumes the liquid. Clinicians should consult with members of the multidisciplinary team and caregivers to identify meaningful goals and treatments.

We focused on treatment of liquid expulsion only (and not solids) because drinking from an open cup and taking bites from a spoon involve different child and therapist responding and different child skill sets. In some cases, re‐presentation may be more challenging to implement with liquids than with solids. For example, it may be difficult for a feeder to quickly collect dispersed liquid to redeposit the liquid into the mouth. Forceful expels that involve the child spraying liquid from the mouth with a burst of air may present a challenge for the feeder when they attempt to collect and re‐present the drink efficiently. For children who display more frequent expels due to the liquid running out of the mouth (e.g., open‐mouth posture), re‐presentation may not be effective, especially if the child does not close their lips to retain fluid inside the oral cavity. In the current study, we did not measure or distinguish between different types of expel consistently or the effects of different types of expel on procedural fidelity; however, this could be an area for future research.

One limitation of the current study was that we did not evaluate other interventions for expulsion that might be perceived as less intensive (e.g., bolus fading) first. Before the study, all participants experienced previous feeding therapies and suffered from severe negative outcomes associated with prolonged refusal of nutritional foods and liquids. Thus, the children's medical teams and caregivers sought more intensive treatment that might result in rapid improvement in their health status. In addition, we conceptualized the general procedure used for meals (i.e., structural components that were in place for preassessments, baseline, and both treatment conditions) as initial antecedent‐based modifications (e.g., presenting small bolus sizes of liquid at evenly paced intervals [30 s]; feeders presenting drinks instead of instructing the child to drink independently; session, volume, and meal time caps). During typical meals at home, caregivers may attempt to present larger portions of liquid (e.g., 8 oz) in potentially more challenging vessels (e.g., large cup). We hypothesized that the initial modifications we made to the mealtime structure could have reduced the response effort associated with drinking relative to previous attempts in the home, but we did not compare those modifications to traditional meals conducted by caregivers.

Future researchers should continue to identify alternatives to extinction‐based interventions, especially for cases in which re‐presentation is ineffective, unnecessary, or not preferred. For example, modifications to liquid viscosity, bolus size, utensils (e.g., syringe), or bolus placements (e.g., Ibañez, Peters, St Paul, et al., 2021) could increase the likelihood of mouth clean and lead to reductions in expel without escape extinction. Children with severe feeding difficulties, like those in the current study, often experience serious health concerns and potentially traumatic events due to their feeding disorder (e.g., frequent hospitalizations, surgeries for and infections resulting from tube insertions; Marsac et al., 2014). Many feeding therapies that are widely promoted or publicized do not result in meaningful improvements for these children (e.g., Peterson et al., 2016). Thus, it is important for researchers to continue to identify effective *and* efficient treatments to produce meaningful and socially valid outcomes.

Another limitation of the current evaluation was that the clinical team did not gather formal social validity measures. The clinical team checked in with caregivers regularly (e.g., daily) and provided caregivers with various treatment options (e.g., antecedent‐based interventions; reinforcement‐based components) along with descriptions and examples of treatment. The clinical team also administered acceptability questionnaires to families that assessed their acceptability of goals, treatment procedures, and outcomes at various points throughout the children's admissions (e.g., early, midway, end). We did not include these social validity data because (a) the questionnaires were related to all treatments (e.g., solids, chewing, caregiver training) and the admission experience, not the study procedures specifically and (b) some questionnaires were not completed in full or were not returned to us by caregivers. Future researchers should gather social validity measures from caregivers, clients, and relevant stakeholders (e.g., feeders, members of the multidisciplinary team).

Diagnosed feeding difficulties represent a complex interaction between behavior and physiology (Rommel et al., 2003). A child's health status depends on consistent intake of calories and hydration and a sufficient food and liquid variety to meet nutritional needs (Manikam & Perman, 2000). For children at risk for failure to thrive, weight loss, or tube placement, dieticians may recommend first targeting a nutritionally complete beverage in treatment (e.g., Pediasure). Thus, researchers should continue to develop treatments for increasing liquid consumption and decreasing expel. Overall, the data in the current evaluation suggest that re‐presentation is an effective intervention for decreasing expulsion. Practitioners who implement re‐presentation should be highly trained and competent to deliver feeding interventions.

## AUTHOR CONTRIBUTIONS

The first author contributed to data analysis, visualization, and writing. The second author contributed to conceptualization, methodology, project administration, data analysis, visualization, supervision, and writing.

## CONFLICT OF INTEREST STATEMENT

The authors declare no financial or nonfinancial interests related to the current study.

## ETHICS APPROVAL

The current study received approval from the university's institutional review board and was conducted in accordance with established ethical guidelines for the treatment of human participants.

## Supporting information


**Data S1** Supporting Information

## Data Availability

Supporting Information A–M includes additional figures with mouth clean and expel per opportunity data. Supporting Information N includes a table with mean inappropriate mealtime behavior per minute across conditions and participants. Additional requests for data can be directed to the corresponding author.
