# Supplementary material for: The role of re‐presentation in the treatment of liquid expulsion
Source: J Appl Behav Anal. 2026 Jan 22;59(1):e70052. doi: 10.1002/jaba.70052 (PMC12825339; doi:10.1002/jaba.70052)
Supplement: Supplementary file 1 — Data S1 Supporting Information [file JABA-59-0-s001.docx]

**SUPPORTING INFORMATION**

**Supporting Information A**

*Percentage of Mouth Clean (Top) and Expel per Opportunity (Bottom) for Mary*

**
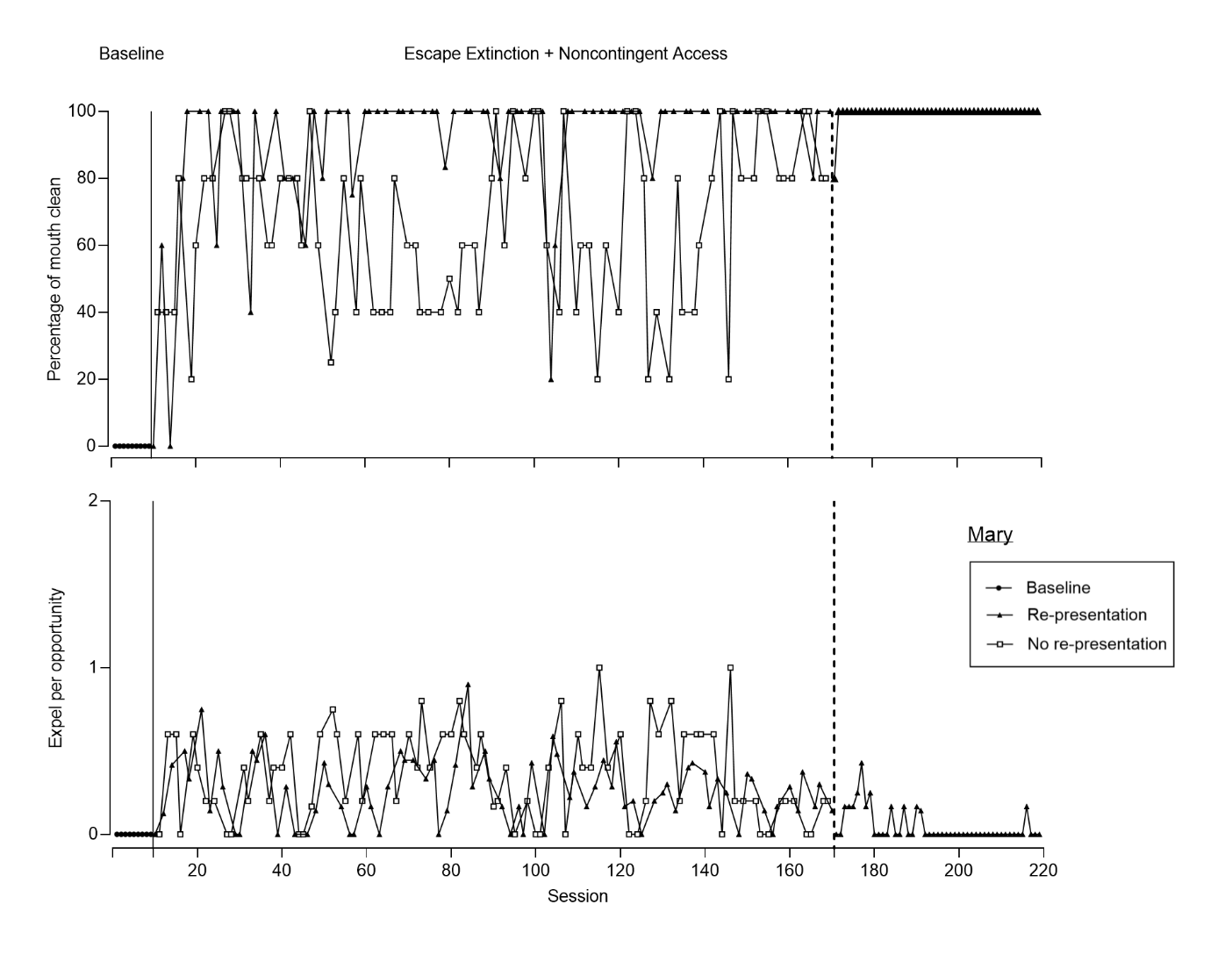
**

**Supporting Information B**

*Percentage of Mouth Clean (Top) and Expel per Opportunity (Bottom) for Blake*


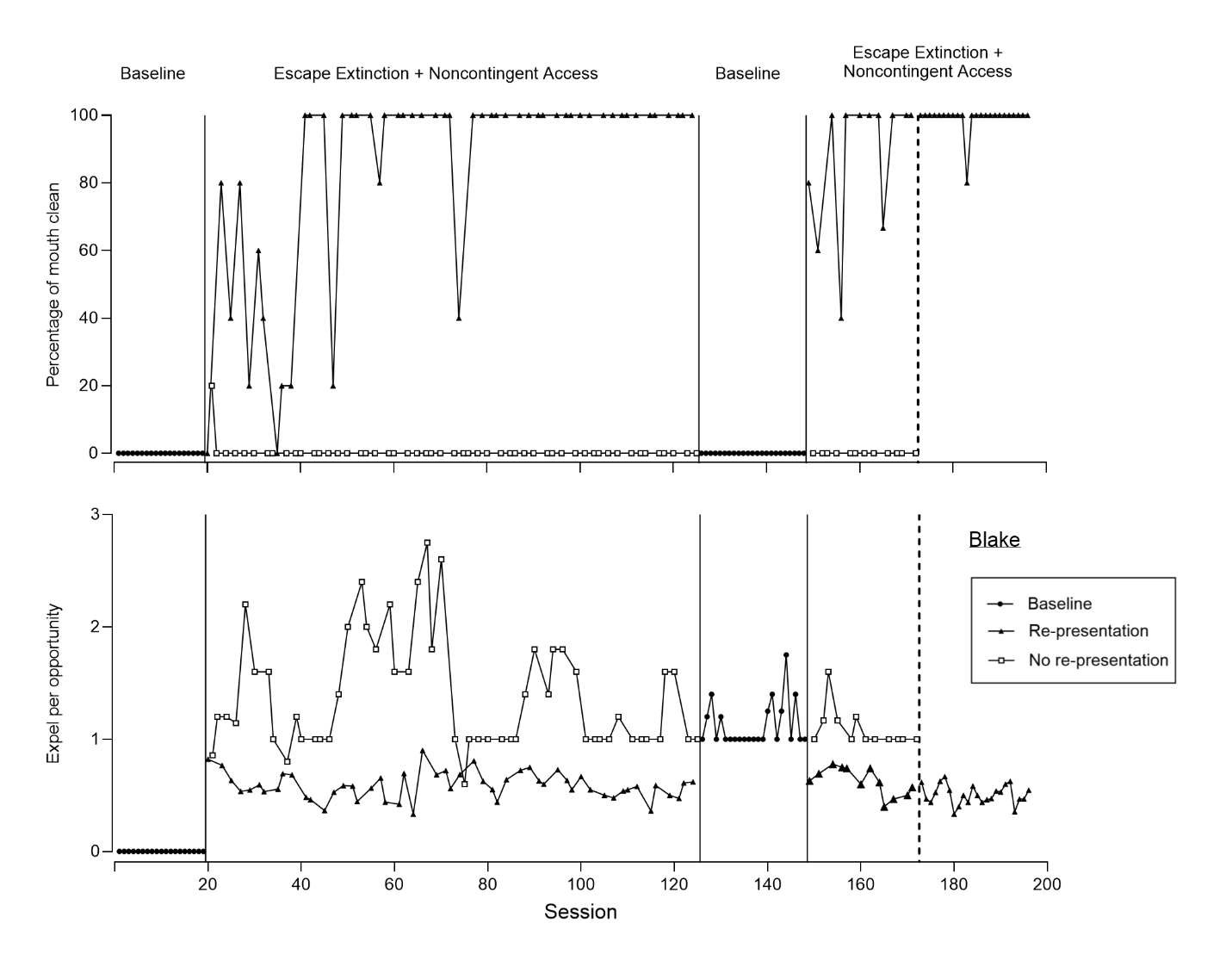


**Supporting Information C**

*Percentage of Mouth Clean (Top) and Expel per Opportunity (Bottom) for Cody*

*
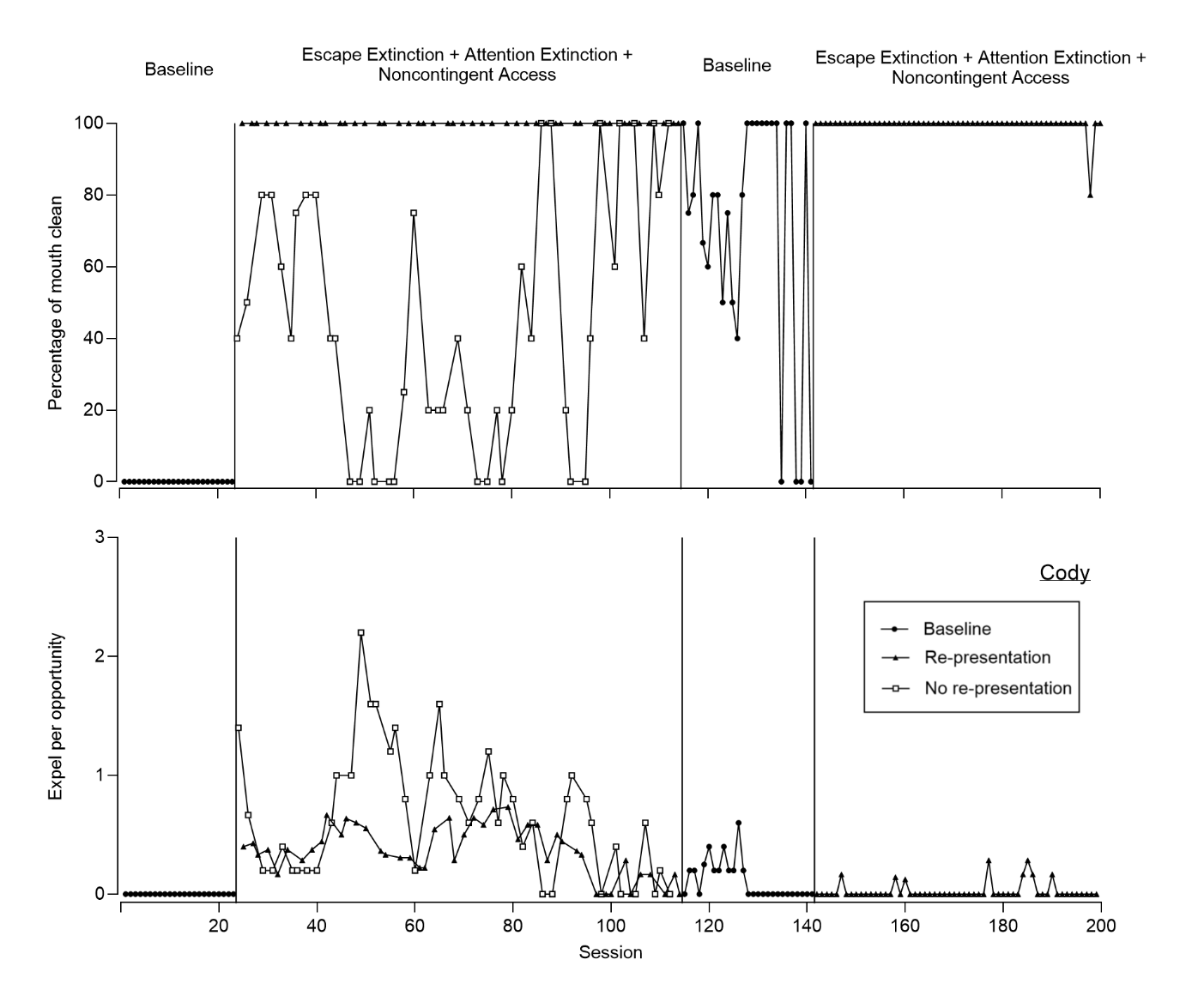
*

**Supporting Information D**

*Percentage of Mouth Clean (Top) and Expel per Opportunity (Bottom) for Dalia*

*
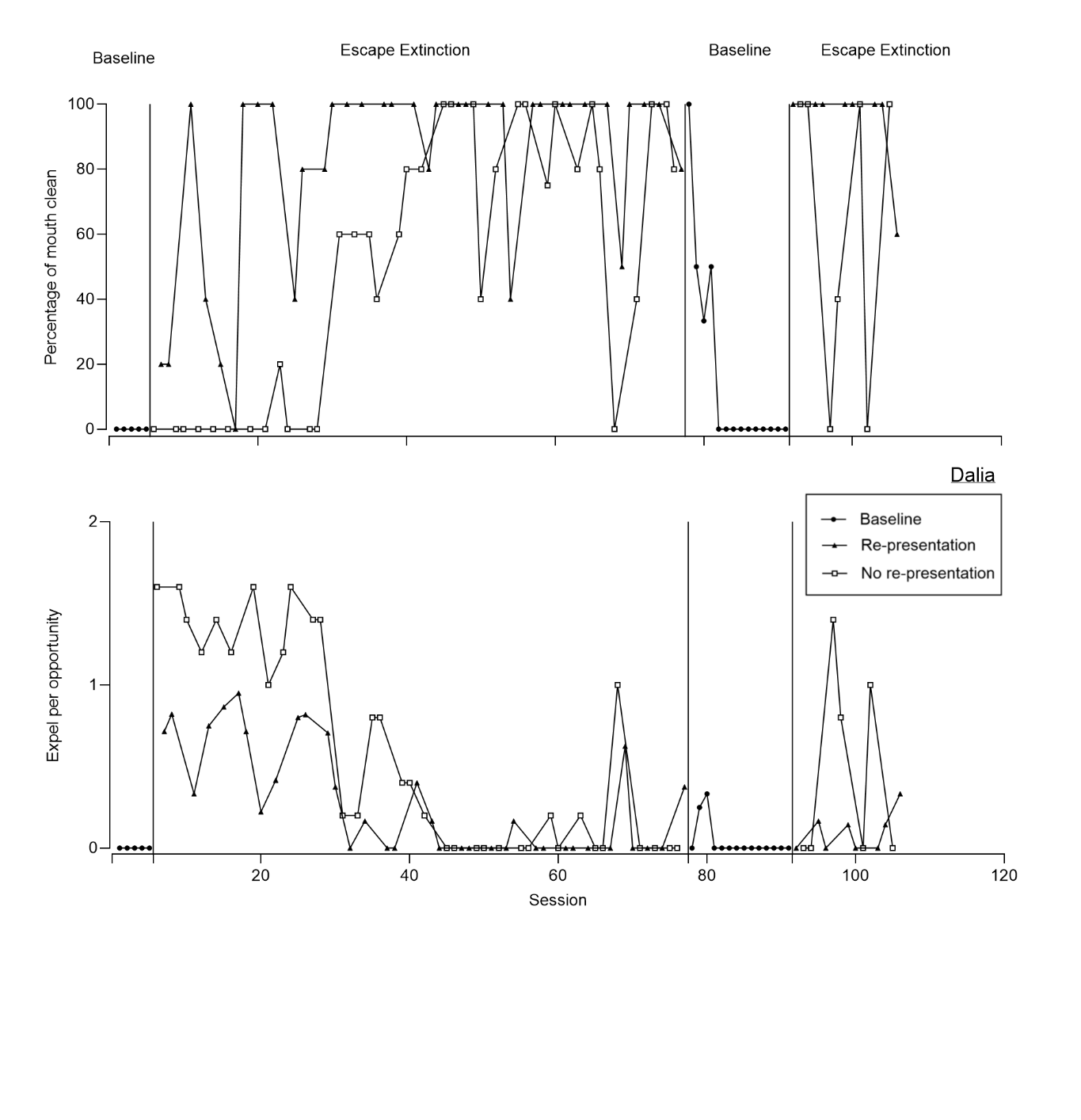
*

**Supporting Information E**

*Percentage of Mouth Clean (Top) and Expel per Opportunity (Bottom) for Fabio*

*
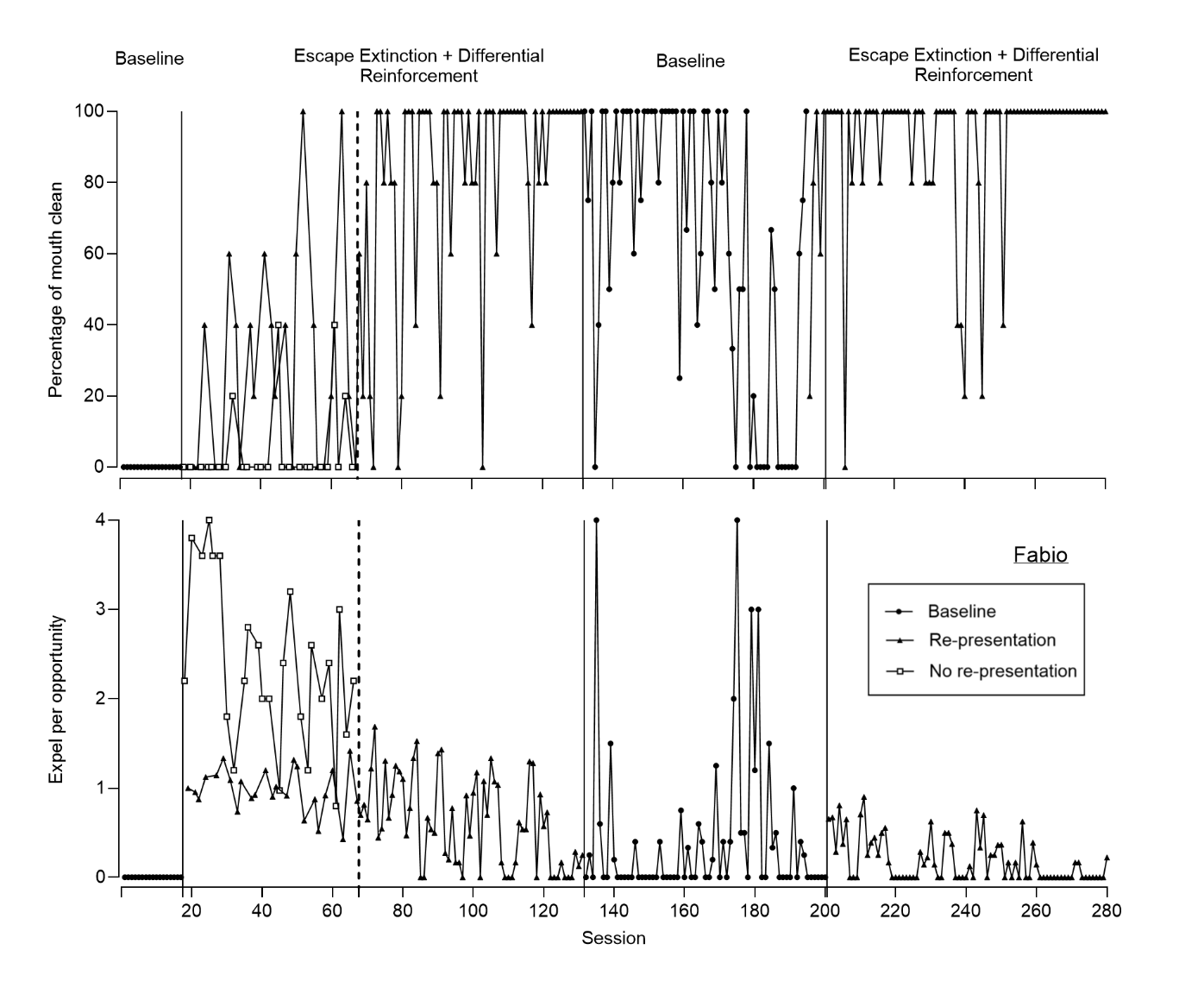
*

**Supporting Information F**

*Percentage of Mouth Clean (Top) and Expel per Opportunity (Bottom) for Levi*

*
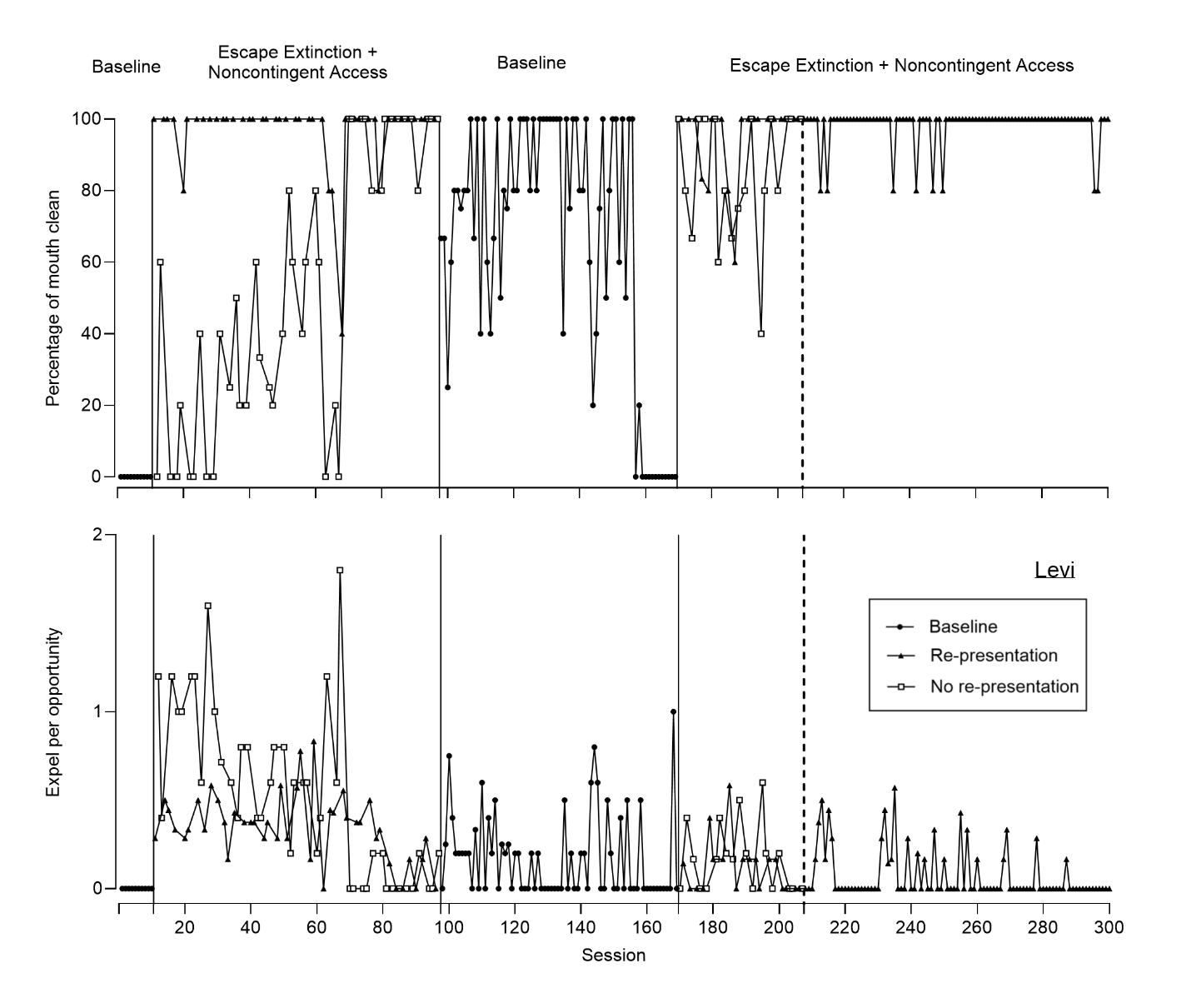
*

**Supporting Information G**

*Percentage of Mouth Clean (Top) and Expel per Opportunity (Bottom) for Liam*

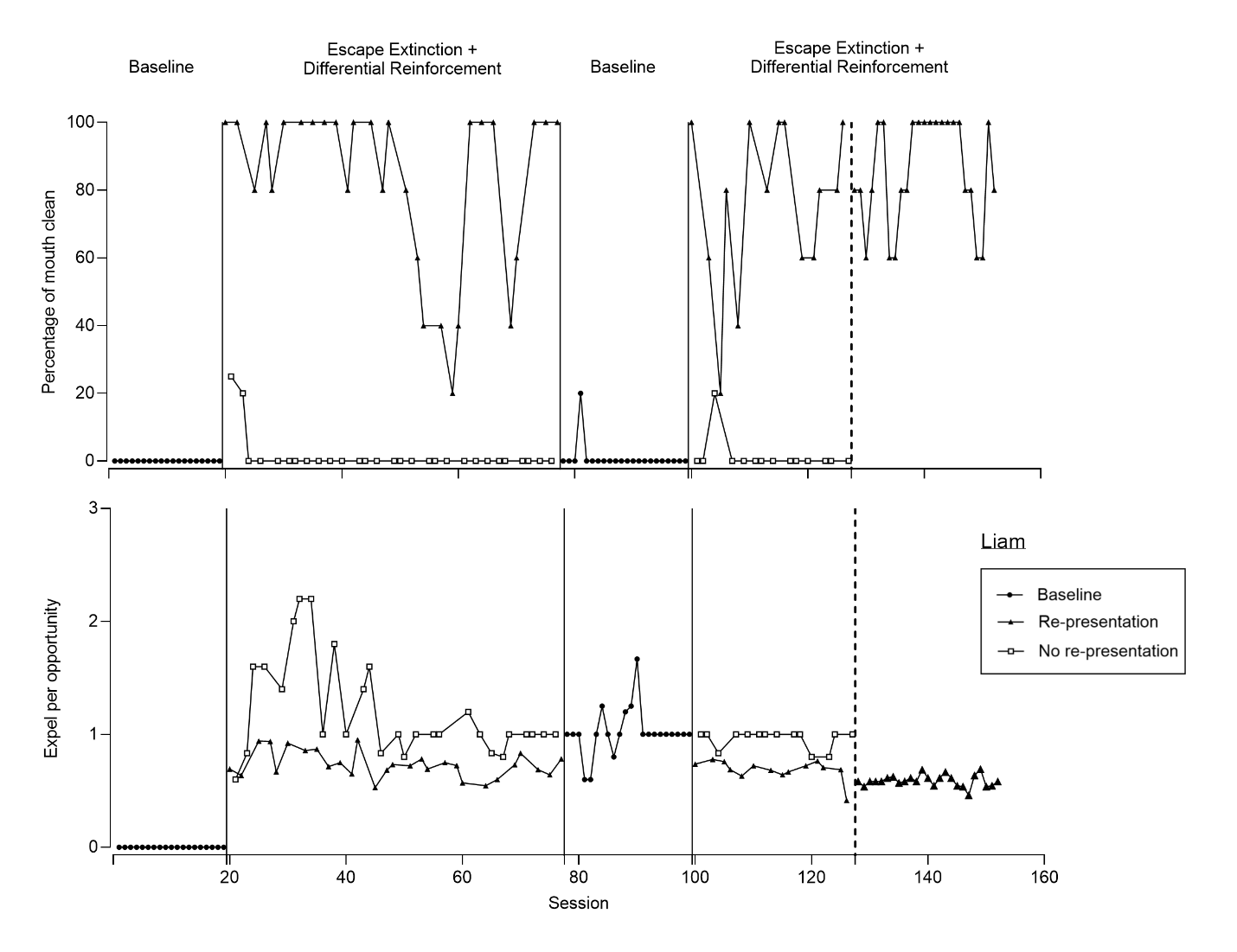


**Supporting Information H**

*Percentage of Mouth Clean (Top) and Expel per Opportunity (Bottom) for Micah*

*
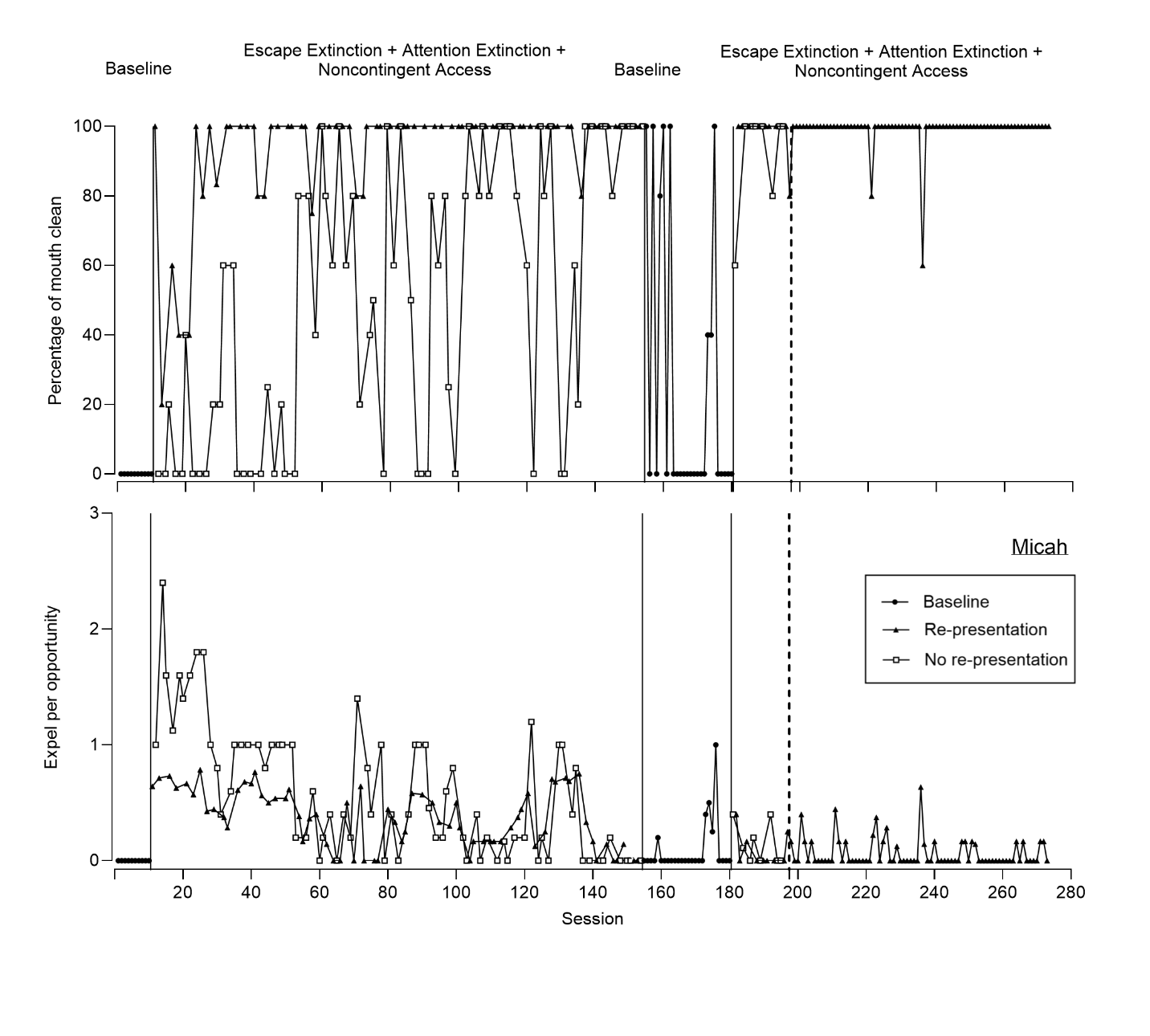
*

**Supporting Information I**

*Percentage of Mouth Clean (Top) and Expel per Opportunity (Bottom) for Sara*

*
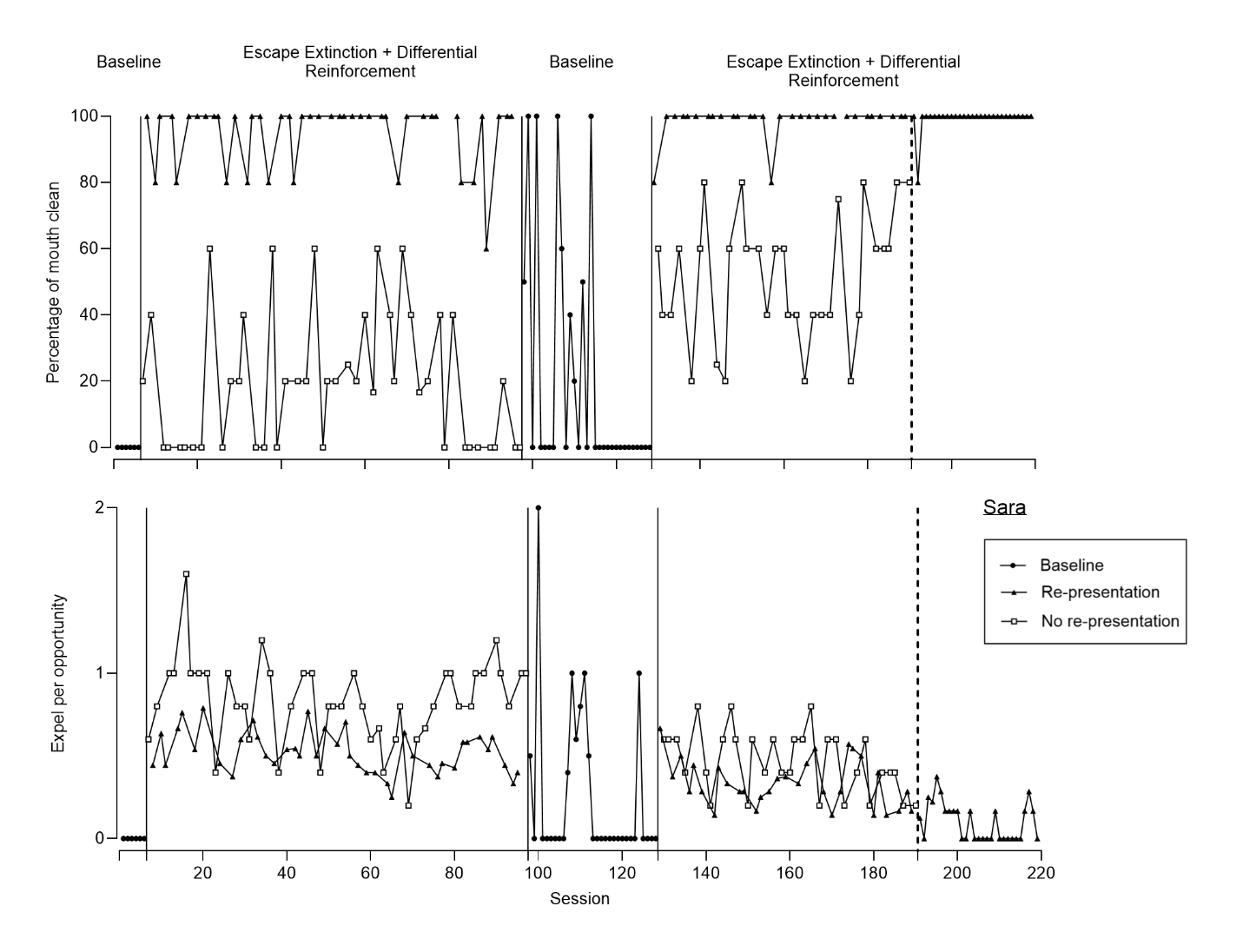
*

**Supporting Information J**

*Percentage of Mouth Clean (Top) and Expel per Opportunity (Bottom) for Siya*

**
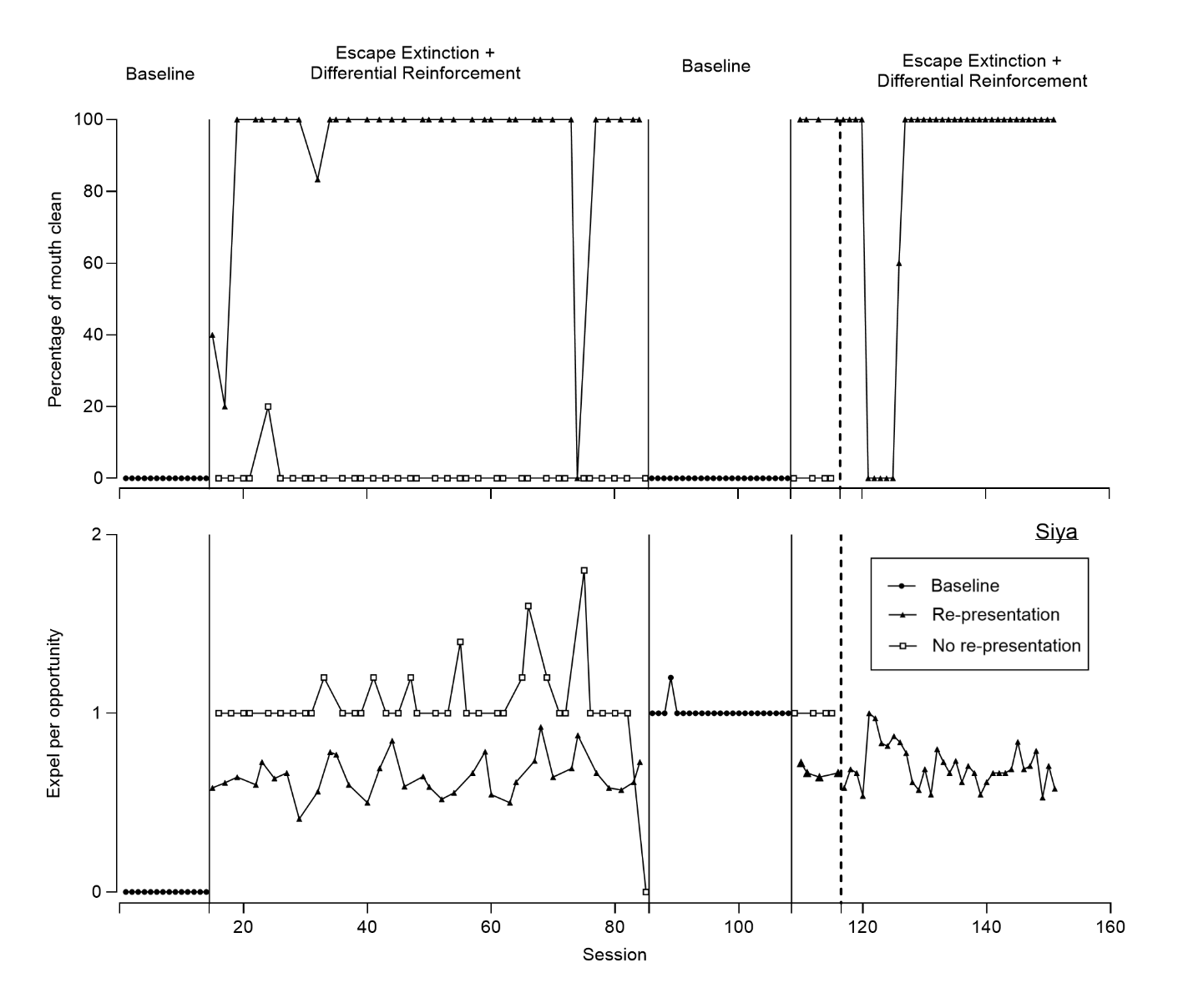
**

**Supporting Information K**

*Percentage of Mouth Clean (Top) and Expel per Opportunity (Bottom) for Maria*

**
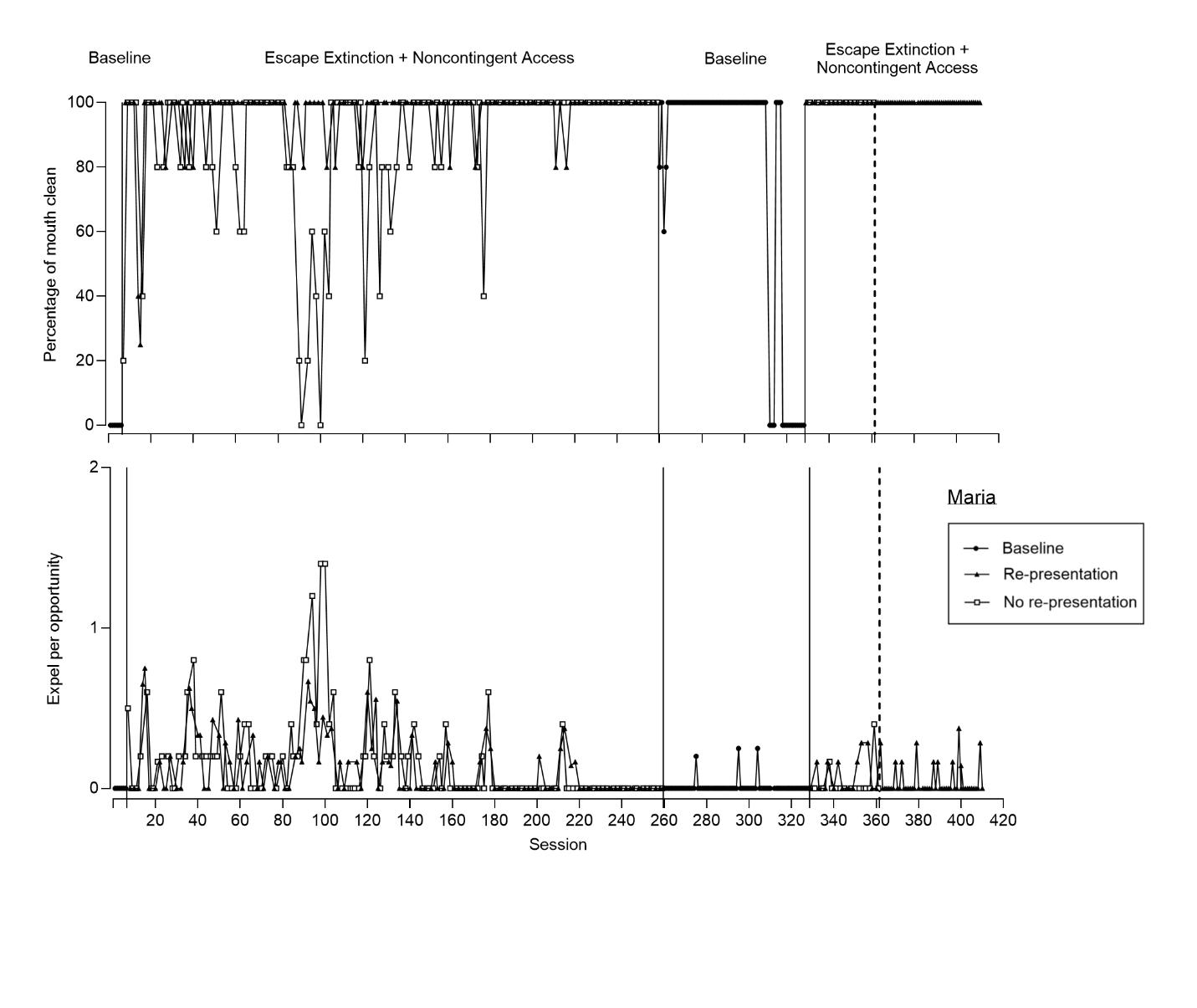
**

**Supporting Information L**

*Percentage of Mouth Clean (Top) and Expel per Opportunity (Bottom) for Kazi*

*
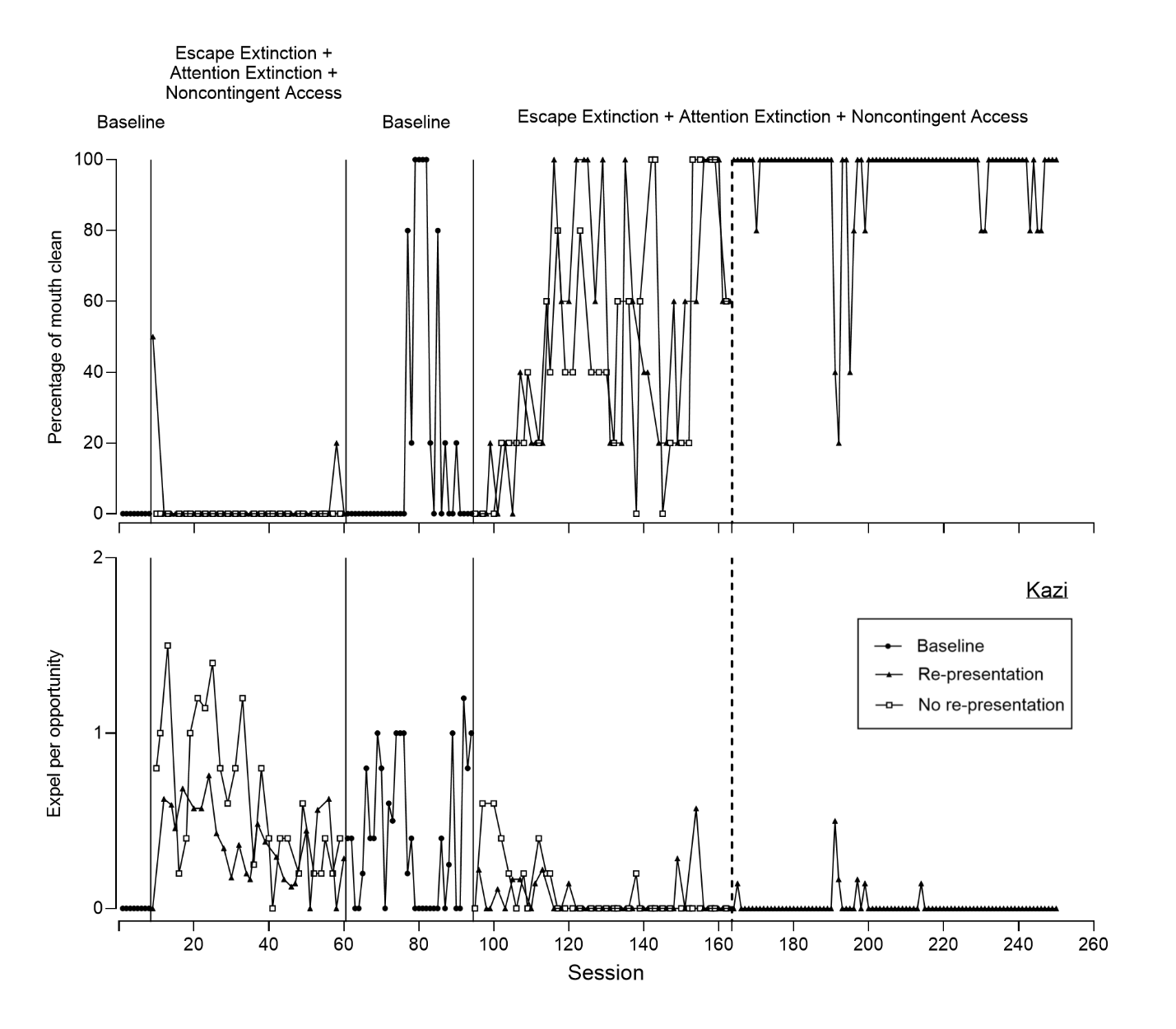
*

**Supporting Information M**

*Percentage of Mouth Clean (Top) and Expel per Opportunity (Bottom) for Cadell*

**
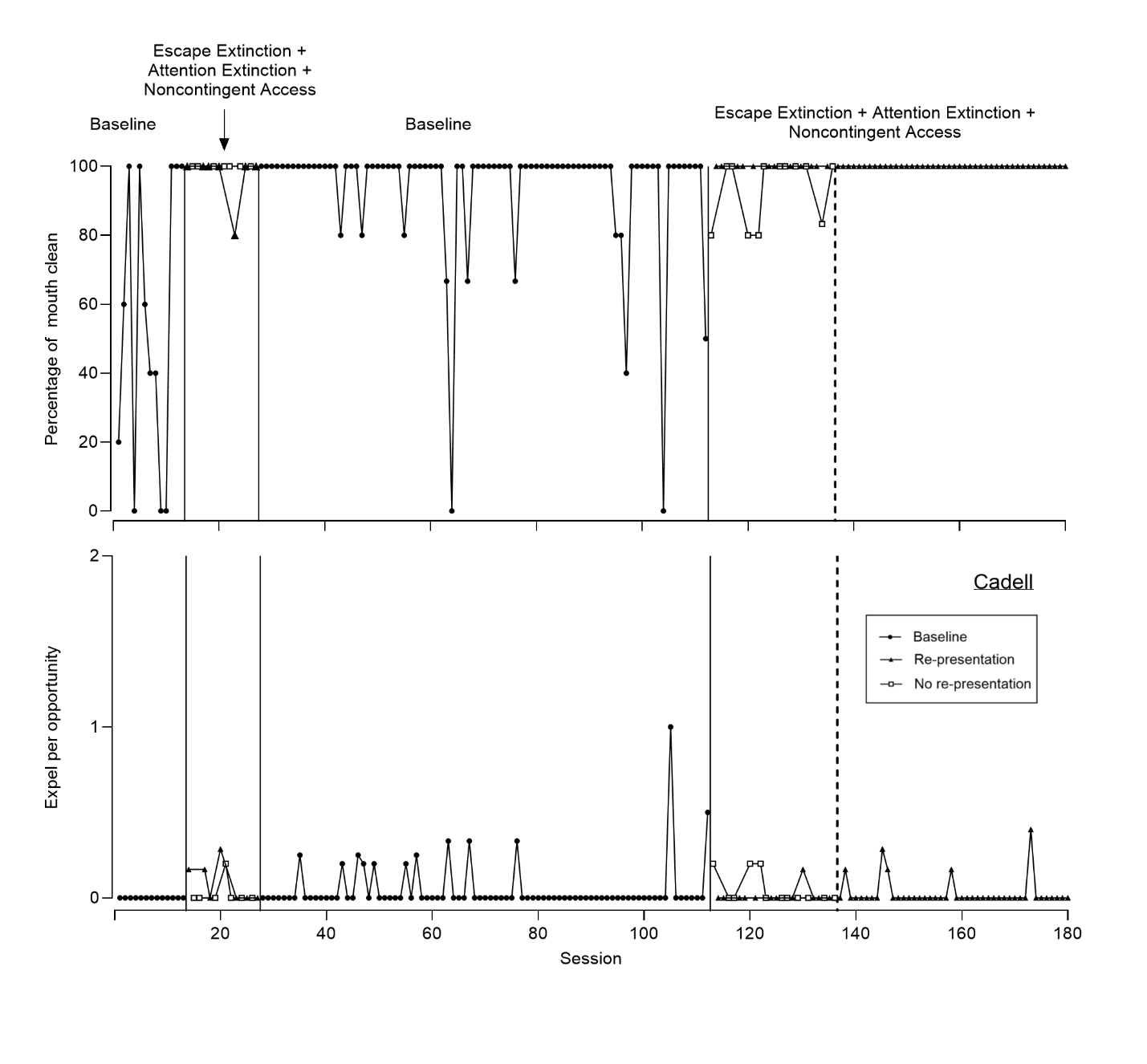
**

**Supporting Information N**

*Mean Inappropriate Mealtime Behavior per Minute Across Conditions*

| **Participant** | **Baseline**  **1** | **Re-presentation 1** | **No Re-presentation**  **1** | **Baseline**  **2** | **Re-presentation**  **2** | **No Re-presentation**  **2** |
| --- | --- | --- | --- | --- | --- | --- |
| Alan | 41.96 | 5.91 | 4.85 | 7.63 | 2.89 | 1.12 |
| Blake | 64.06 | 3.52 | 5.51 | 1.89 | 0.46 | 1.93 |
| Cody | 29.65 | 8.63 | 11.07 | 13.58 | 1.86 | N/A |
| Dalia | 21.35 | 3.68 | 6.30 | 28.15 | 0 | 0 |
| Fabio | 38.17 | 4.90 | 2.49 | 11.18 | 4.90 | N/A |
| Levi | 35.96 | 2.38 | 1.65 | 6.75 | 2.22 | 1.65 |
| Liam | 26.90 | 2.98 | 5.48 | 2.68 | 2.85 | 1.54 |
| Micah | 42.88 | 6.64 | 6.43 | 37.66 | 2.73 | 5.52 |
| Sara | 31.01 | 4.05 | 5.17 | 4.64 | 3.82 | 2.87 |
| Siya | 17.46 | 9.05 | 11.60 | 0.17 | 2.65 | 0 |
| Simon | 43.47 | 3.13 | 5.10 | 1.76 | 1.39 | 1.24 |
| Maria | 37.10 | 3.43 | 3.55 | 10.57 | 2.85 | 1.23 |
| Kazi | Kazi | 38.37 | 6.75 | 6.27 | 0.69 | 0.59 |
| Ava | 39.81 | 6.62 | 7.82 | 47.59 | 1.27 | 0.69 |
| Cadell | 19.45 | 0 | 1.89 | 3.37 | 4.46 | 0.84 |
| Jay | 29.38 | 10.57 | 10.08 | 0.28 | 0.34 | 0.94 |
| Ella | 5.1 | 2.09 | 0.23 | N/A | N/A | N/A |

*Note.* N/A = not applicable.
